# Integrating compositional and functional content to describe vaginal microbiomes in health and disease

**DOI:** 10.1186/s40168-023-01692-x

**Published:** 2023-11-30

**Authors:** Johanna B. Holm, Michael T. France, Pawel Gajer, Bing Ma, Rebecca M. Brotman, Michelle Shardell, Larry Forney, Jacques Ravel

**Affiliations:** 1 Institute for Genome Sciences, University of Maryland School of Medicine, Baltimore, MD USA; 2 Department of Microbiology and Immunology, University of Maryland School of Medicine, Baltimore, MD USA; 3 Department of Epidemiology and Public Health, University of Maryland School of Medicine, Baltimore, MD USA; 4 Department of Biological Sciences, University of Idaho, Moscow, ID USA

**Keywords:** Vaginal microbiome, Genital health, Metagenome, Sequencing, Bacterial vaginosis

## Abstract

**Background:**

A *Lactobacillus-*dominated vaginal microbiome provides the first line of defense against adverse genital tract health outcomes. However, there is limited understanding of the mechanisms by which the vaginal microbiome modulates protection, as prior work mostly described its composition through morphologic assessment and marker gene sequencing methods that do not capture functional information. To address this gap, we developed metagenomic community state types (mgCSTs) which use metagenomic sequences to describe and define vaginal microbiomes based on both composition and functional potential.

**Results:**

MgCSTs are categories of microbiomes classified using taxonomy and the functional potential encoded in their metagenomes. MgCSTs reflect unique combinations of metagenomic subspecies (mgSs), which are assemblages of bacterial strains of the same species, within a microbiome. We demonstrate that mgCSTs are associated with demographics such as age and race, as well as vaginal pH and Gram stain assessment of vaginal smears. Importantly, these associations varied between mgCSTs predominated by the same bacterial species. A subset of mgCSTs, including three of the six predominated by *Gardnerella* *vaginalis* mgSs, as well as mgSs of *L. iners*, were associated with a greater likelihood of bacterial vaginosis diagnosed by Amsel clinical criteria. This *L. iners* mgSs, among other functional features, encoded enhanced genetic capabilities for epithelial cell attachment that could facilitate cytotoxin-mediated cell lysis. Finally, we report a mgSs and mgCST classifier for which source code is provided and may be adapted for use by the microbiome research community.

**Conclusions:**

MgCSTs are a novel and easily implemented approach to reduce the dimension of complex metagenomic datasets while maintaining their functional uniqueness. MgCSTs enable the investigation of multiple strains of the same species and the functional diversity in that species. Future investigations of functional diversity may be key to unraveling the pathways by which the vaginal microbiome modulates the protection of the genital tract. Importantly, our findings support the hypothesis that functional differences between vaginal microbiomes, including those that may look compositionally similar, are critical considerations in vaginal health. Ultimately, mgCSTs may lead to novel hypotheses concerning the role of the vaginal microbiome in promoting health and disease, and identify targets for novel prognostic, diagnostic, and therapeutic strategies to improve women’s genital health.

Video Abstract

**Supplementary Information:**

The online version contains supplementary material available at 10.1186/s40168-023-01692-x.

## Background

The vaginal microbiome plays a vital role in gynecological and reproductive health. *Lactobacillus* predominated vaginal microbiota constitute the first line of defense against infection. Protective mechanisms include lactic acid production by *Lactobacillus* spp., which acidifies the vaginal microenvironment and elicits anti-inflammatory effects [1–4]. This environment wards off non-indigenous organisms, including causative agents of sexually transmitted infections (STIs) like HIV, and bacteria associated with bacterial vaginosis (BV) [5–7]. However, vaginal *Lactobacillus* spp. are functionally diverse. For example, *L. crispatus*, and *L. gasseri* are capable of producing both the D- and L-isomers of lactic acid, *L. jensenii* produces only the D-isomer, and *L. iners* only the L-isomer [4, 8]. These key features have implications for susceptibilities to pathogens [9, 10].

The vaginal microbiota has been previously shown to cluster into community state types (CSTs) that reflect differences in bacterial species composition and abundance [1, 11]. *Lactobacillus* spp. predominate four of the five CSTs (CST I: *L. crispatus*; CST II: *L. gasseri*; CST III: *L. iners*, CST V: *L. jensenii*). In contrast, CST IV communities are characterized by a paucity of lactobacilli and the presence of a diverse array of anaerobes such as *G. vaginalis* and “*Ca.* Lachnocurva vaginae”. CST IV is found, albeit not exclusively, during episodes of BV, a condition associated with an increased risk of sexually transmitted infections, including HIV, as well as preterm birth and other gynecological and obstetric adverse outcomes [12–20]. BV is clinically defined by observing 3 of 4 Amsel’s criteria (Amsel-BV; vaginal pH > 4.5, abnormal discharge, and on wet mount, presence of clue cells and fishy odor with 10% KOH) [21]. Patients presenting with symptoms and satisfying Amsel’s criteria (symptomatic Amsel-BV) are treated with antibiotics, however, efficacy is poor, and recurrence is common [21–24]. In research settings, BV is often defined by scoring Gram-stained vaginal smears (Nugent-BV) [25] or molecular typing of bacterial composition by sequencing marker genes (molecular-BV) [26]. There is no definition of BV that relies on both the composition and functional potential of the microbiome.

Species-level composition of the vaginal microbiota may not suffice to accurately capture associations between the vaginal microbiome and outcomes of interest because functional differences exist between strains of the same species. For example, in the skin microbiome, strains of *Staphylococcus aureus* or *Streptococcus pyogenes* elicit different acute immune responses [27]. Similarly, genomic and functional analyses of *Lactobacillus rhamnosus* strains demonstrate distinct adaptations to specific niches (for example, the gut versus the oral cavity) [28]. While functional differences likely exist between strains of the same species in the vaginal microbiota, metagenomic studies show that combinations of multiple strains co-exist within a single vaginal microbiome [29, 30]. These strain assemblages have been defined as metagenomic subspecies or mgSs [29], and are important to consider as they potentially impact the functional diversity and resilience of a species in a microbiome. Determining the mechanistic consequences and health outcomes associated with metagenomic subspecies may improve the precision of risk estimates and interventions.

To integrate the taxonomic composition and functional potential of vaginal microbiomes, we developed metagenomic community state types (mgCSTs). MgCSTs are composed of unique combinations of mgSs. We developed and validated a two-step classifier that assigns metagenomic subspecies and mgCSTs and is designed to work in concert with the vaginal non-redundant gene database, VIRGO [29]. This classifier may facilitate reproducibility and comparisons across studies.

## Results

### Metagenomic community state types (mgCST) of the vaginal microbiome

**Fig. 1:**
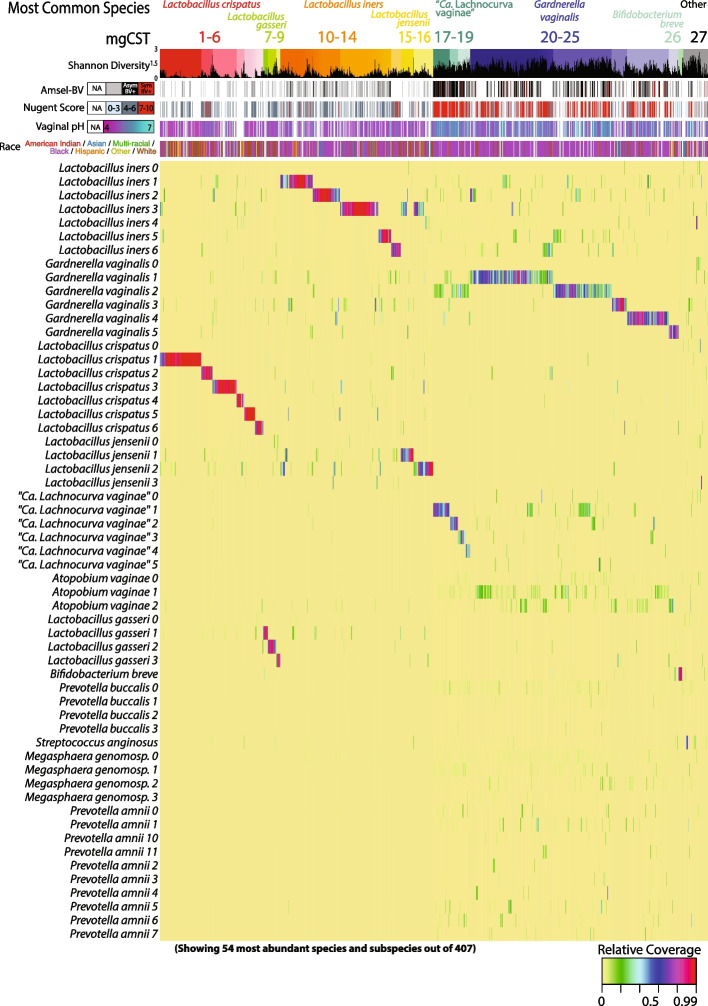
Vaginal Metagenomic Community State Types (mgCSTs). Using 1890 metagenomic samples, 27 vaginal metagenomic Community State Types (mgCSTs) were identified: mgCSTs 1–16 are predominated by metagenomic subspecies of *Lactobacillus* spp., mgCSTs 17–19 by metagenomic subspecies of “*Ca*. Lachnocurva vaginae”, mgCSTs 20–25 by metagenomic subspecies of the genus *Gardnerella*, and mgCST 27 contains samples without a predominant metagenomic subspecies

We evaluated the within-species bacterial genomic diversity in 1890 vaginal metagenomes of reproductive-age participants from 1024 mostly North American women (98.7% of samples) (Table 1). Vaginal metagenomes derived from five cohort studies as well as metagenomes generated to build the vaginal non-redundant gene database (VIRGO, [29]) were used to construct mgCSTs (see “Methods” section). In total, 135 metagenomic subspecies (mgSs) from 28 species were identified by hierarchical clustering of species-specific gene presence/absence profiles (Table S1). Subsequent hierarchical clustering of samples based on mgSs compositional data produced 27 mgCSTs (Table 2). Cluster stability was ≥ 0.75 for most mgCSTs except for “*Ca.* Lachnocurva vaginae” mgCSTs 17–19 where cluster stability was ≥ 0.55 (Table 2). MgCSTs consisted of mgSs from commonly observed vaginal species including *L. crispatus* (mgCST 1–6, 19% of samples), *L. gasseri* (mgCST 7–9, 3% of samples), *L. iners* (mgCST 10–14, 23% of samples), *L. jensenii* (mgCST 15 and 16, 4.6% of samples), “*Ca.* Lachnocurva vaginae” (mgCST 17–19, 7.5% of samples), *Gardnerella* (mgCST 20–25, 36.3% of samples) and *Bifidobacterium breve* (mgCST 26, 0.74% of samples) (Fig. 1). MgCST 27 (5.5% of samples) contained less-common species such as *Streptococcus anginosus* or had no predominant taxon. In the case of *Gardnerella* genomospecies other than *G. vaginalis*, there were fewer genes in each metagenome than would be expected in a full genome (data not shown). Thus, there is at present insufficient genetic evidence of multiple strains of genomospecies in a single metagenome. Therefore, mgSs were created using all *Gardnerella* genes and referred to as *Gardnerella vaginalis*. MgCST 2 (*n* = 39 samples from 26 women), mgCST 14 (*n* = 34 samples from 25 women), and mgCST 21 (*n* = 37 samples from 21 women) were only comprised of samples from reproductive-aged women in Alabama enrolled in the UMB-HMP cohort (Table 2). Metagenomic CSTs expand amplicon-based CSTs as multiple mgCSTs are predominated by the same species, but different mgSs of that species (Figure S1, Table 2).

**Table 1:** Characteristics of study participants

Categories	Number of women with category-specific data (*N* = 1024)	Number of samples with category-specific data (*N* = 1890)
Metagenomic data source	1017	1890
UMB-HMP	124 (12.2%)	515 (27.2%)
Li et al.	44 (4.3%)	44 (2.3%)
LSVF	585 (57.5%)	653 (34.6%)
NIH-HMP	76 (7.5%)	174 (9.2%)
VMRC	40 (3.9%)	162 (8.6%)
VIRGO	148 (14.6%)	342 (18.1%)
Age (years)	897	1623
15–20	283 (31.5%)	410 (25.3%)
21–25	229 (25.5%)	436 (26.9%)
26–30	188 (21.0%)	362 (22.3%)
31–35	102 (11.4%)	223 (13.7%)
36–40	65 (7.2%)	125 (7.7%)
41–45	30 (3.3%)	67 (4.1%)
Race	858	1441
Asian	54 (6.3%)	66 (4.6%)
Black or African-American	610 (71.1%)	968 (67.2%)
Hispanic or Latino	19 (2.2%)	47 (3.3%)
Other	6 (0.7%)	9 (0.6%)
White or Caucasian	169 (19.7%)	351 (24.4%)
Nugent category	968	1623
0–3	469 (48.5%)	931 (57.4%)
4–6	194 (20.0%)	255 (15.7%)
7–10	305 (31.5%)	437 (26.9%)
Vaginal pH category	874	1362
Low (pH < 4.5)	273 (31.2%)	491 (36.0%)
High (pH ≥ 4.5)	601 (68.8%)	871 (64.0%)
Amsel-BV diagnosis	627	673
Positive	289 (46.1%)	308 (45.8%)
Negative	338 (53.9%)	365 (54.2%)
Symptomatic Amsel-BV	289	308
Asymptomatic	253 (87.5%)	271 (88.0%)
Symptomatic	36 (12.5%)	37 (12.0%)

For each data category (age, race, etc.), the total number of women and samples are noted. Percentages represent the proportions of women or samples within each category. Some women contributed multiple samples

Vaginal Non-redundant Gene Database (VIRGO, virgo.igs.umaryland.edu) [29], the University of Maryland Baltimore Human Microbiome Project (UMB-HMP, PRJNA208535, PRJNA575586, PRJNA797778), the National Institutes of Health Human Microbiome Project (NIH-HMP, phs000228), Li et al. [31] (PRJEB24147), the Longitudinal Study of Vaginal Flora and Incident STI (LSVF, dbGaP project phs002367)

**Table 2:** Metagenomic community state types (mgCSTs) of the vaginal microbiome are dominated by different metagenomic subspecies

MgCST	Most frequently detected mgSs	Most abundant mgSs	Number of samples	Number of women	Cluster stability (100 bootstraps)	Median Shannon Index	Number of samples from metagenomic data source					
							UMB-HMP	Li et al.	LSVF	HMP	VIRGO	VMRC
1	*Lactobacillus crispatus 1*	*Lactobacillus crispatus 1*	143	79	0.977	0.17	20	2	21	63	15	22
2	*Lactobacillus crispatus 2*	*Lactobacillus crispatus 2*	39	26	0.965	0.47	39	0	0	0	0	0
3	*Lactobacillus crispatus 3*	*Lactobacillus crispatus 3*	83	51	0.912	0.28	9	2	15	14	22	21
4	*Lactobacillus crispatus 4*	*Lactobacillus crispatus 4*	27	12	0.972	0.39	1	1	0	0	3	22
5	*Lactobacillus crispatus 5*	*Lactobacillus crispatus 5*	37	27	0.960	0.11	1	0	19	16	1	0
6	*Lactobacillus crispatus 6*	*Lactobacillus crispatus 6*	28	13	0.982	0.69	12	0	5	2	9	0
7	*Lactobacillus gasseri 1*	*Lactobacillus gasseri 1*	16	8	0.786	0.5	0	0	1	8	1	6
8	*Lactobacillus gasseri 2*	*Lactobacillus gasseri 2*	29	17	0.945	0.73	15	0	8	0	1	5
9	*Lactobacillus gasseri 3*	*Lactobacillus gasseri 3*	14	5	0.425	0.89	6	0	0	2	0	6
10	*Lactobacillus iners 1*	*Lactobacillus iners 1*	113	76	0.912	0.7	24	0	40	2	10	37
11	*Lactobacillus iners 2*	*Lactobacillus iners 2*	95	79	0.862	0.53	11	7	47	0	28	2
12	*Lactobacillus iners 3*	*Lactobacillus iners 3*	131	92	0.927	0.44	9	10	45	19	42	6
13	*Lactobacillus iners 5*	*Lactobacillus iners 5*	45	41	0.912	0.57	1	0	29	1	13	1
14	*Lactobacillus iners 6*	*Lactobacillus iners 6*	34	25	0.945	0.8	34	0	0	0	0	0
15	*Lactobacillus jensenii 1*	*Lactobacillus jensenii 1*	44	28	0.888	0.77	8	1	3	10	18	4
16	*Lactobacillus jensenii 2*	*Lactobacillus jensenii 2*	67	39	0.859	0.71	8	0	15	15	13	16
17	"*Ca.* Lachnocurva vaginae" 1	"*Ca.* Lachnocurva vaginae" 1	58	57	0.552	1.48	3	0	51	1	3	0
18	"*Ca.* Lachnocurva vaginae" 2	"*Ca.* Lachnocurva vaginae" 2	28	27	0.574	1.57	0	0	27	0	1	0
19	"*Ca.* Lachnocurva vaginae" 3	"*Ca.* Lachnocurva vaginae" 3	43	36	0.574	1.91	7	0	27	0	9	0
20	*Gardnerella vaginalis 1*	*Gardnerella vaginalis 1*	250	171	0.799	1.62	90	2	98	2	38	20
21	*Gardnerella vaginalis 1*	*Gardnerella vaginalis 1*	37	21	0.542	1.97	37	0	0	0	0	0
22	*Gardnerella vaginalis 2*	*Prevotella amnii 4*	202	159	0.607	1.79	30	0	91	3	67	11
23	*Gardnerella vaginalis 3*	*Gardnerella vaginalis 3*	53	42	0.862	0.88	18	5	15	2	5	8
24	*Gardnerella vaginalis 4*	*Gardnerella vaginalis 4*	145	106	0.959	1.21	44	0	64	6	24	7
25	*Gardnerella vaginalis 5*	*Gardnerella vaginalis 5*	34	17	0.983	0.83	11	1	9	6	2	5
26	*Bifidobacterium breve*	*Bifidobacterium breve*	16	11	0.750	0.9	5	0	1	0	8	2
27	*Bifidobacterium dentium*	*Enterococcus faecalis 3*	87	76	0.750	1.78	23	13	22	2	9	18

*UMB-HMP* University of Maryland Baltimore-Human Microbiome Project, *Li et al. *[32] PRJEB24147; *LSVF* Longitudinal Study of the Vaginal Flora, *HMP* Human Microbiome Project, *VIRGO* virgo.igs.umaryland.edu, *VMRC* Vaginal Microbiome Research Consoritum

### Vaginal mgCSTs and demographics

#### Race and age

**Fig. 2:**
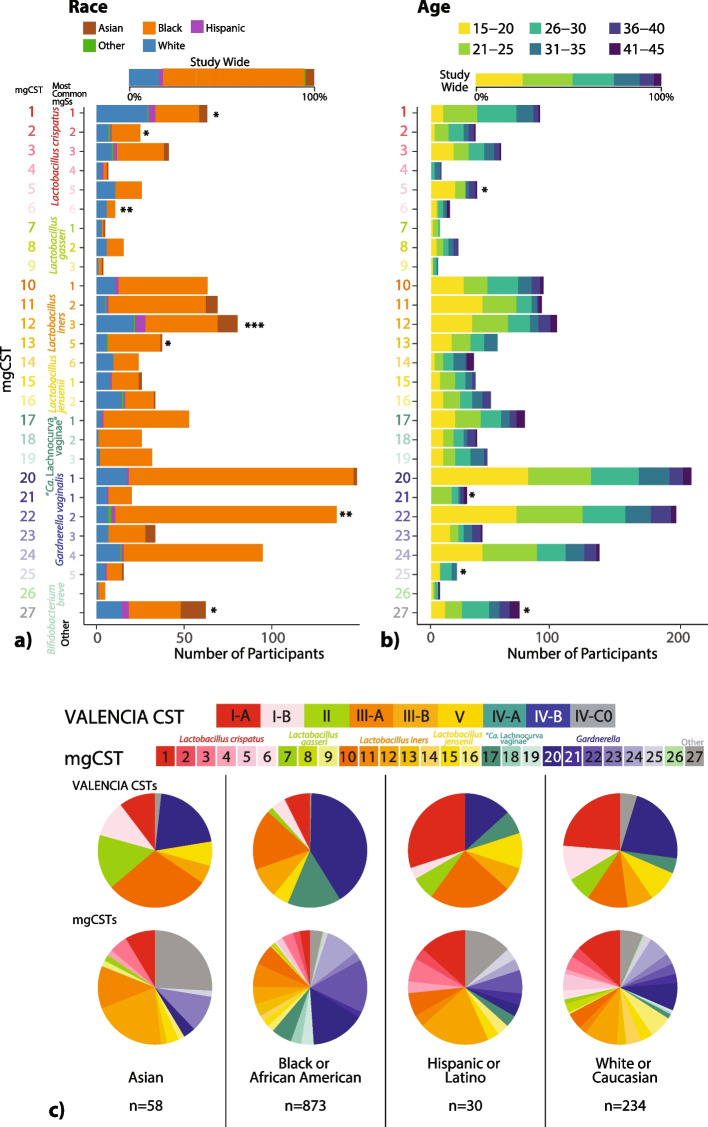
MgCSTs were associated with self-reported race (**a**, *n* = 1441) and age categories (**b**, *n* = 1623) Within-mgCST distributions were compared to study-wide distributions (*p < 0.05, **p < 0.01, ***p < 0.001). **c** The distribution of mgCSTs differs by race. Vaginal microbiomes from black women have the smallest proportion of *L. crispatus* mgCSTs. “*Ca.* Lachnocurva vaginae” mgCSTs 17–19 were absent from Asian vaginal microbiomes in this study, as were *L. iners* mgCST 10 and 14

Race information was available for 1441 samples reported by 858 women. Most women self-identified as either Black (71%) or White (20%), and the remainder as Asian (6.3%), Hispanic (2.2%), or other (< 1%) (Table 1). Age was also reported for 1623 samples from 897 individuals and ranged from 15 to 45 years old. After adjusting for between-cohort heterogeneity, certain races and age categories were associated with mgCSTs (Fig. 2). The vaginal microbiomes of Black women were more likely to be classified as *Gardnerella* mgCST 22 (*p* = 0.0006) and least likely to be in *L. crispatus* mgCST 1 (*p* = 0.005) as compared with microbiomes for other races (Table S2). Microbiomes classified as mgCST 6 were more likely to be from White women than other races (*p* = 0.002). *L. iners* mgCST 12 was most common among Hispanic and Asian women (*p* = 0.0001), and *L. iners* mgCSTs 10 and 14 were not detected in Asian women (Fig. 2c). MgCSTs predominated by “*Ca.* Lachnocurva vaginae” (mgCSTs 17–19) were primarily comprised of samples from Black women and did not contain any samples from Asian women, though 19% of samples (*n* = 12) from Asian women were in CST IV (Fig. 2c). Instead, these samples were assigned to *L. iners* mgCST 12 (*n* = 1) *Gardnerella* mgCSTs 20 or 23 (*n* = 8) or were predominated by *Streptococcus anginosus* (mgCST 27, *n* = 3). In mgCST 27, women were less likely to be Black (*p* = 0.01) and more likely to be in the oldest age category (41–45, *p* = 0.04) as compared with other mgCSTs.

#### Nugent scores and vaginal pH

**Fig. 3:**
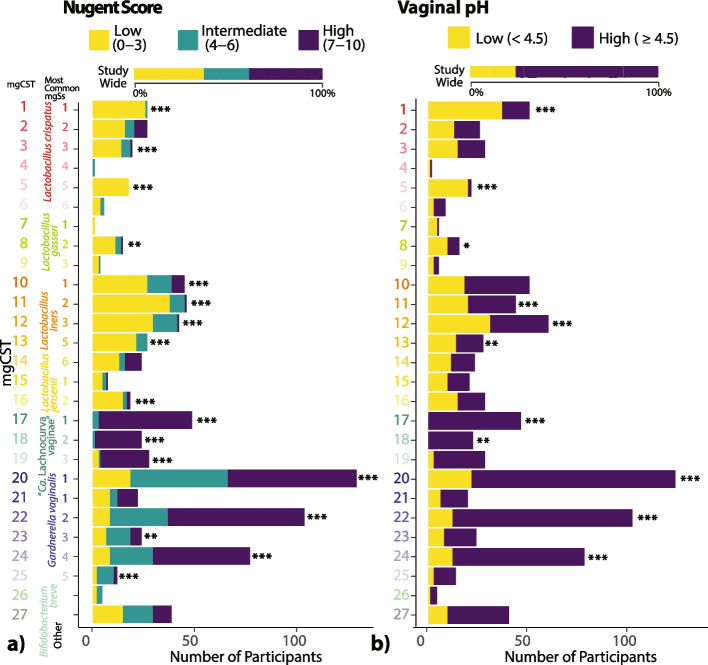
MgCSTs are associated with Nugent Scores (**a**, *n* = 968) and vaginal pH categories (**b**, *n* = 979). Within-mgCST distributions were compared to study-wide distributions (*p < 0.05, **p < 0.01, ***p < 0.001)

Of the 968 women for which Nugent scores were available, 48% had low Nugent scores (0–3), 20% had intermediate scores (4–6), and 32% had high scores (7–10) (Table 1). Vaginal pH was also available for 979 women and of these 31% had low pH < 4.5, and 69% had high pH ≥ 4.5 (Table 1). Both Nugent score and vaginal pH were associated with mgCSTs after adjusting for between-cohort heterogeneity (Fig. 3). Of all *L. crispatus* mgCSTs, mgCST 2 had the most representation of different Nugent categories, with 61%, 14%, and 25% of samples having low, intermediate, or high Nugent scores, respectively (Fig. 3a). Communities predominant in “*Ca.* Lachnocurva vaginae” mgCSTs 17, 18, and 19 had the highest percentages of high Nugent scores (7–10), (94%, 96%, and 87% of samples, respectively); and these mgCSTs were also associated with high vaginal pH (*p* = 6.3 e^−7^, Fig. 3b). Notably, intermediate Nugent scores were common among *Gardnerella* *vaginalis* predominated mgCSTs, especially in mgCSTs 25 (69% of samples).

#### Amsel-BV and vaginal symptoms

**Fig. 4:**
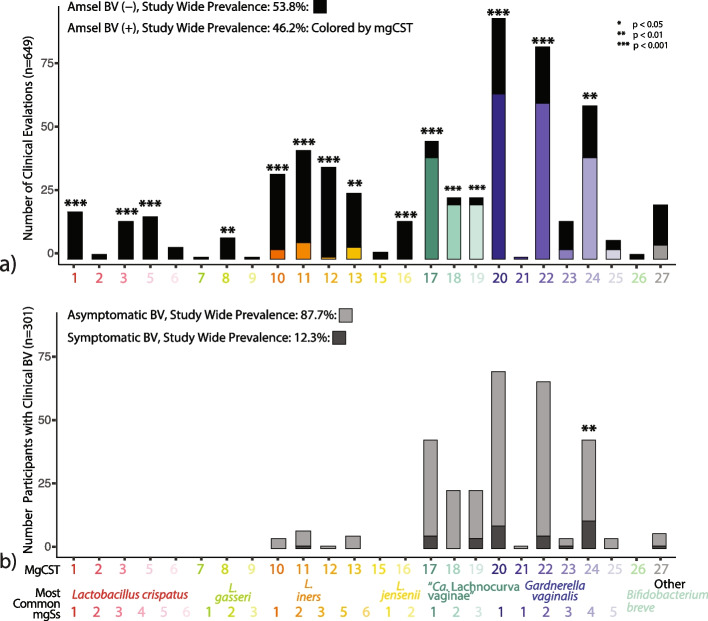
Clinically diagnosed Amsel bacterial vaginosis (**a**) and symptomatic Amsel bacterial vaginosis (**b**) associated with mgCSTs. **a** Within each mgCST, the total number of clinical evaluations per mgCST is indicated. The black bars indicate the proportion of negative Amsel-BV diagnoses, and the colored bars indicate the positive Amsel-BV diagnoses. Within-mgCST proportions were statistically compared to the study-wide proportions of Amsel-BV diagnoses. **b** Of positive Amsel-BV diagnoses in **a**, the proportions of asymptomatic (light gray) and symptomatic (dark gray) Amsel-BV diagnoses are shown within each mgCST. Within-mgCST proportions were statistically compared to the study-wide proportions of asymptomatic to symptomatic Amsel-BV diagnoses. (*p < 0.05, **p < 0.01, ***p < 0.001)

Of 627 women, each with a vaginal sample and same-day clinical examination data (*n* = 607 from LSVF cohort, *n* = 20 from HMP cohort), 40.3% had asymptomatic Amsel-BV and 5.5% had symptomatic Amsel-BV diagnoses. Twelve percent of Amsel-BV cases were symptomatic. Diagnosis of Amsel-BV was associated with mgCSTs (Fig. 4a). There were no Amsel-BV diagnoses in mgCSTs predominated by *L. crispatus, L. jensenii*, or *L. gasseri*. *L. iners* predominated mgCSTs 10–13 were negatively associated Amsel-BV diagnoses (*p* = 9.6e^−4^) but contained some positive Amsel-BV diagnoses in mgCSTs 10, 11, and 13 (11%, 15%, 18% of women, respectively) (Fig. 4a and Table S2). *L. iners* mgCST 12 contained only a single (asymptomatic) positive Amsel-BV diagnosis out of 39 women. Women with “*Ca.* Lachnocurva vaginae” mgCSTs 17–19 were more likely to have been diagnosed with Amsel-BV (87%, 88%, and 89%, respectively, *p* = 1.8e^−5^). *Gardnerella* predominated mgCSTs 20, 22, and 24 also had significantly more positive Amsel-BV diagnoses than the study-wide proportion (69%, 73%, and 66%, respectively, *p* = 1.5e^−3^), while 75% of *Gardnerella* predominated mgCST 23 samples were Amsel-BV negative (*p* = 0.09). MgCST 24 contained significantly more symptomatic cases than expected (26% of 43 individuals, *p* = 0.008, Fig. 4b, Table S2). Though not statistically significant, *“Ca.* Lachnocurva vaginae” mgCST 19 also may have a higher-than-expected proportion of symptomatic Amsel-BV cases (17.4%).

### Functional potential of mgCSTs and metagenomic subspecies

#### *L. crispatus* mgCSTs differ by species diversity, stability, and the potential to produce D-lactic acid

**Fig. 5:**
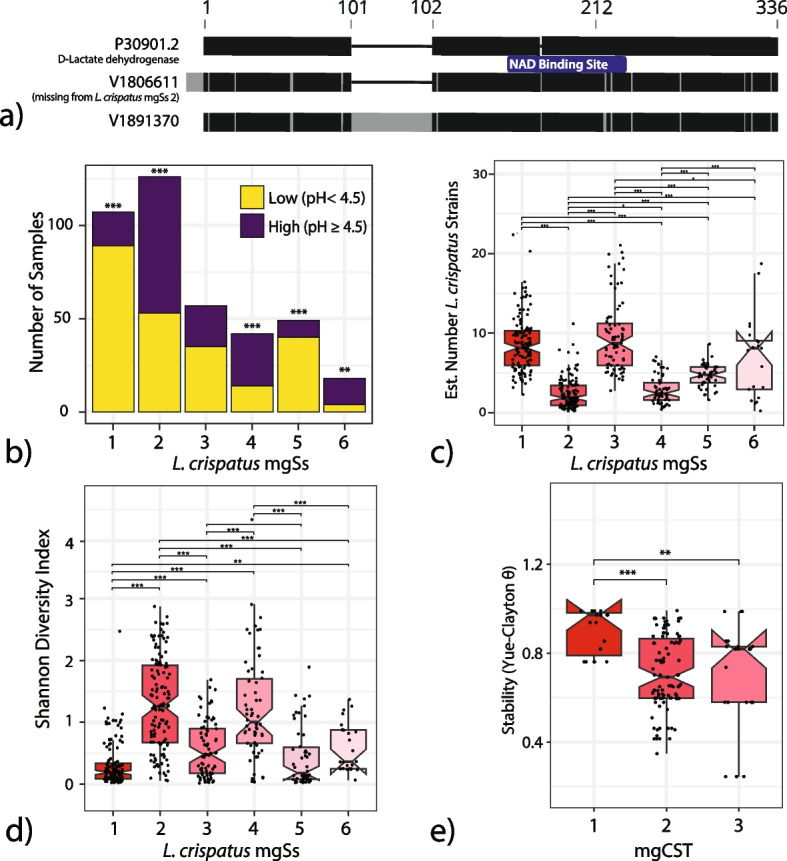
**a** D-lactate dehydrogenase orthologs in VIRGO compared to functionally validated reference, P30901.2. The presence of D-lactate dehydrogenase orthologs differs by *L. crispatus* mgSs with mgSs 2 missing V1806611 which is 96% identical to the functionally validated ortholog P30901.2. **b** The proportion of samples with low vs. high vaginal pH differed by the *L. crispatus* mgSs present (reference was based on the proportions among samples containing any *L. crispatus* mgSs: 40.7% low and 59.4% high). **c** The estimated number of *L. crispatus* strains differed by the *L. crispatus* mgSs present. **d** Shannon diversity of the vaginal microbiome differed by the *L. crispatus* mgSs present. **e** Microbiome stability differed by *L. crispatus* mgCST

*L. crispatus* is known to produce both L- and D-lactic acid, which acidifies the vaginal environment and confers protective properties [4, 10, 33, 34]. Metagenomic analyses revealed differences among *L. crispatus* mgSs. First, VIRGO identified two L- and two D-lactate dehydrogenase genes in *L. crispatus*. All genes were present in *L. crispatus* mgSs except for mgSs 2. MgSs 2 was missing a D-lactate dehydrogenase gene (V1806611) that has a 96.1% identity to a functionally validated ortholog, P30901.2 (Fig. 5a) [35]. The other D-lactate dehydrogenase, V1891370, is found in all *L. crispatus* mgSs but only 82.4% identical to P30901.2 because it contains a 55 aa insertion after V101 (position in P30901.2) and a point mutation at position 218 (D218Y) located within a NAD binding site domain, the functional consequences of which are unknown. Because V1806611 is most similar to P30901.2, its absence may influence the production of D-lactic acid. Second, in mgSs 2, 4, and 6 60–70% of samples were significantly more likely to be from the high vaginal pH category compared to 41% of samples with any *L. crispatus* mgSs (Fig. 5b). Third, mgSs 2 had significantly fewer estimated numbers of *L. crispatus* strains compared to other *L. crispatus* mgSs (Fig. 5c). Fourth, mgSs 2 and 4 were on average more compositionally diverse than other mgSs (Fig. 5d). Lastly, mgCST 1 (dominated by mgSs 1) was significantly more longitudinally stable than mgCST 2 or 3 as defined using Yue-Clayton’s θ (Fig. 5e). Overall, these differences reveal important genetic and functional differences among vaginal microbiomes primarily comprised of *L. crispatus* which could be important in understanding the role of *L. crispatus* in the vaginal microbiome.

#### *L. iners* metagenomic subspecies are associated with Amsel-BV diagnoses

**Fig. 6:**
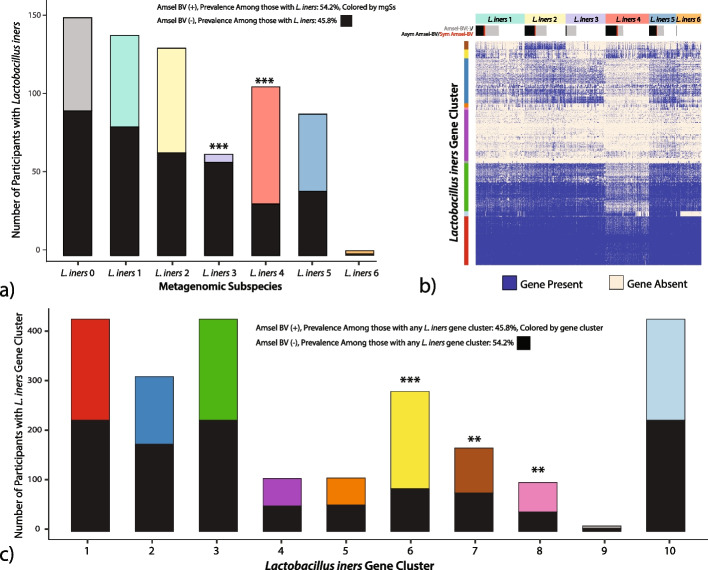
**a** The total number of clinical evaluations per *L. iners* mgSs are indicated. The black bars indicate the proportion of negative Amsel-BV diagnoses, and the colored bars indicate the positive Amsel-BV diagnoses. **b** Gene presence map represents gene content of *L. iners* mgSs (columns) and *L. iners* gene clusters (rows). **c** The total number of clinical evaluations per *L. iners* gene cluster is indicated. The black bars indicate the proportion of negative Amsel-BV diagnoses, and the colored bars indicate the positive Amsel-BV diagnoses. Within-gene group proportions were statistically compared to the study-wide proportions of Amsel-BV for any samples containing a *L. iners* gene cluster. (*p < 0.05, **p < 0.01, ***p < 0.001)

The role of *L. iners* in the vaginal microbiome is not fully understood because it has been implicated in both healthy and BV states [36]. Sixty-five percent of samples containing *L. iners* mgSs 4 were positive Amsel-BV cases which is significantly greater than the proportion of cases harboring any *L. iners* mgSs (45.8%, *p* < 0.001, Fig. 6a). Conversely, *L. iners* mgSs 3, which predominates mgCST 12, was associated with negative Amsel-BV diagnoses (86% Amsel-BV negative, *p* < 0.0001). *L. iners* is represented by six mgSs of which 5 predominated an mgCST; *L. iners* mgSs 4 did not (Fig. 1). Instead, *L. iners* mgSs 4 was present in relatively lower abundances (median 1.2%, IQR 1.9%) in 257 microbiomes from BV-like mgCSTs 16, 17, and 18, 19, and 24.

We next evaluated if genes from *L. iners* were associated with Amsel-BV. Most samples in *L. iners* mgSs 4 contained genes from cluster 6 (yellow gene cluster, Fig. 6b). There were significantly more positive Amsel-BV diagnoses among participants containing *L. iners* gene cluster 6 (69.4%, *p* < 0.001), 7 (53.9%, *p* = 0.004), or 8 (60.2%, *p* = 0.036) compared to samples containing any other *L. iners* gene cluster (45.8%, Fig. 6c). Gene products unique to *L. iners* gene cluster 6 had significant similarity to virulence factors that could contribute to *L. iners* ability to thrive in dynamic vaginal states. Such factors include serine/threonine-protein kinases (STPKs), SHIRT domains known as “periscope proteins” which regulate bacterial cell surface interactions related to host colonization [37], CRISPR-*cas,* β-lactamase and multidrug resistance (MATE), and bacteriocin exporters (Table S3). Gene products in cluster 7 included ParM, which plays a vital role in plasmid segregation, pre-protein translocation, membrane anchoring (SecA, SecY, sortase), defense mechanism beta-lytic metallopeptidase, and mucin-binding and internalin proteins. In *Listeria monocytogenes*, internalin A mediates adhesion to epithelial cells and host cell invasion [38]. Phage-like proteins in gene group 8 suggest the presence of mobile elements. The presence of the highly conserved *L. iners* pore-forming cytolysin, inerolysin [39], did not differ by mgSs.

#### Diversity of *Gardnerella* genomospecies differs by mgCST

**Fig. 7:**
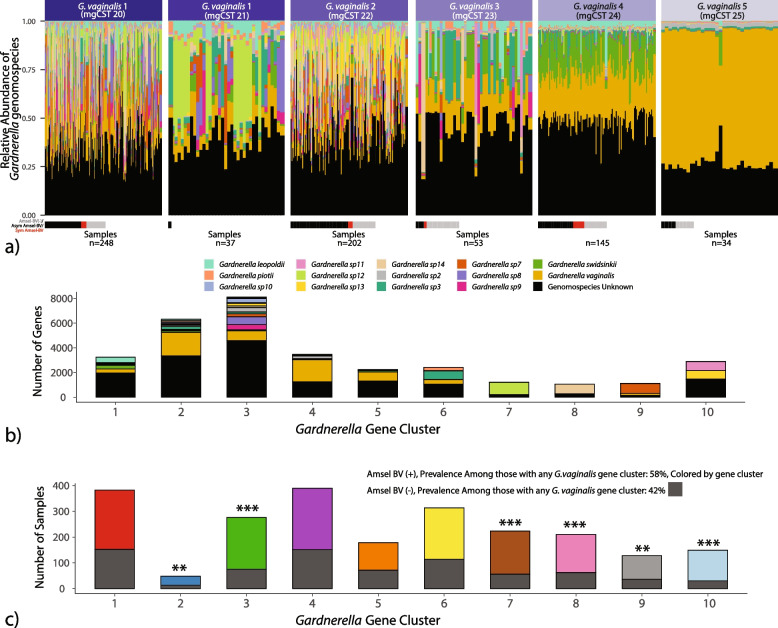
The proportions of *Gardnerella* genomospecies in a sample differ by *Gardnerella* mgSs (**a**). For samples with Amsel-BV evaluations, Amsel-BV status is indicated below each sample (column). **b** Gene clusters of *Gardnerella* contain genes attributed to a variety of *Gardnerella* genomospecies (as indicated by colored bars, black bars indicate unknown genomospecies). **c** The total number of clinical evaluations per *Gardnerella* gene cluster is indicated. The dark gray bars indicate the proportion of negative Amsel-BV diagnoses, and the colored bars indicate the positive Amsel-BV diagnoses. Within-gene group proportions were statistically compared to the study-wide proportions of Amsel-BV for any samples containing a *Gardnerella* gene cluster. (*p < 0.05, **p < 0.01, ***p < 0.001)

As previously mentioned, positive Amsel-BV diagnoses were common in *Gardnerella* mgCSTs 20, 22, and 24, while mgCST 23 contained relatively more Amsel-BV negative samples than positive Amsel-BV (Fig. 4). Symptomatic BV cases were more common in mgCST 24 than in all other mgCSTs*.* By mapping the available genomes of various *Gardnerella* genomospecies [40] to VIRGO, we determined that each *Gardnerella* mgSs consists of a unique combination of *Gardnerella* genomospecies (Fig. 7a). Compared to other *Gardnerella* mgCSTs, mgCSTs 20–22 contain a greater number of *Gardnerella* genomospecies than mgCSTs 23–25. MgCST 24 samples are predominated by *Gardnerella* mgSs 4 and largely consist of *G. swidsinkii* and *G. vaginalis* genes. This suggests the diversity and types of *Gardnerella* genomospecies may be important determinants of the pathogenicity of mgCSTs. *Gardnerella* gene clusters contained different proportions of *Gardnerella* genomospecies (Fig. 7b). Certain gene clusters were associated with Amsel-BV diagnoses, especially gene cluster 10 (Fig. 7c), which is primarily comprised of genes from *Gardnerella* sp 11 and sp 13 (Fig. 7b) and encodes a vaginolysin (V1099403), a hemolysin (V1099398), the muralytic enzyme precursor Rpf2 (V1385511), a vancomycin resistance protein (V1099665), and glycogen debranching enzymes pullulanase (V1313000) and oligo-16-glucosidase (V1195401).

### Automated classification of mgCSTs using random forest models

Random forest models were built for each of the 135 mgSs identified and used to perform mgSs assignments (see “Methods” section). There was good concordance between mgSs assigned by hierarchical clustering of Jensen-Shannon distances and random forest-based assignments with κ > 0.8 for most species (Figure S3a). Ten-fold cross-validation of the classifier revealed the misclassification error for mgSs assignment ranged from 0 to 30% (Figure S2c mgSs). The error estimates for most major vaginal taxa were near or less than 10%, with *L. gasseri* having the lowest (2.2%). *L. iners* consistently provided high misclassification error estimates (20%) regardless of attempts to fine-tune the model and was likely the result of high genetic homogeneity between and/or heterogeneity within *L. iners* mgSs. Following the assignment of mgSs, mgCSTs were assigned using the nearest centroid classification method, as previously used for vaginal taxonomy-based community state-type assignments [11]. We observed good concordance between mgCSTs assigned by hierarchical clustering of Jensen-Shannon distances and nearest centroid-based assignments (κ = 0.78, Figure S3b). Ten-fold cross validation of centroid classification revealed the mean classification error was 9.6%, with some mgCSTs classified more accurately than others (Figure S4).

**Fig. 8:**
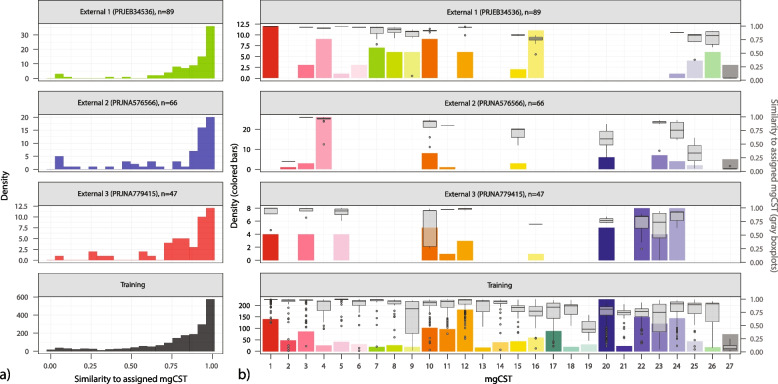
Three external metagenomic datasets were processed with VIRGO [29] and assigned mgCSTs using the mgCST classifier. **a** Most samples were statistically similar to the reference centroid of the assigned mgCST. **b** Samples in each dataset were distributed across mgCSTs. In all cases, the lowest similarity scores were observed in mgCST 27

Three external, publicly available metagenomic datasets illustrate the generalizability of the mgCSTs assignment (Fig. 8). Most samples produced similarity scores > 0.5 indicating high similarity to the reference centroid of the assigned mgCST (Fig. 8a). Samples in each dataset were distributed across mgCSTs (Fig. 8b, primary *y*-axis). In all datasets, the lowest similarity scores were observed in mgCST 27 (Fig. 8b, secondary *y*-axis).

The source code for the mgCST classifier is an R script and is available at https://github.com/ravel-lab/mgCST-classifier and uses direct outputs from VIRGO.

## Discussion

Recent findings that motivated the development of mgCST classification are that multiple strains of the same species are commonly observed in the vaginal microbiome [29], and that samples can be clustered into metagenomic subspecies defined by unique strain combinations represented by species-specific gene sets, and thus unique sets of functions. These critical observations led us to conceptualize a vaginal microbiome classification based on their mgSs compositions and abundance and thus defined by both species’ composition and functions, *i.e.*, metagenomic community state types. MgCSTs describe vaginal microbiomes through a new lens, one that includes both compositional and functional dimensions.

*L. iners-*predominated vaginal microbiota have been associated with an increased risk of experiencing bacterial vaginosis (BV) [41, 42]. Longitudinal observational prospective studies support this conclusion and present several critical findings: (1) *L. iners* is often detected at low to medium abundances during episodes of BV [43], (2) *L. iners* commonly dominate the vaginal microbiota after metronidazole treatment for BV [44], and, (3) *L. iners* predominated vaginal microbiota are more prevalent prior to incidence of BV [41, 43]. However, *L. iners* is not always associated with an increased risk of negative outcomes and its role in the vaginal microbiome has been widely debated [36, 45–48]. MgCST classification provides insight into the contradiction to prevailing dogma regarding L. iners and increased risk of BV. L. iners mgSs 4 was associated with Amsel-BV, while L. iners mgSs 3 (predominates mgCST 12) was significantly associated with negative BV diagnoses. This is the first evidence of genetically distinct combinations of L. iners strains (mgSs) in healthy versus BV-like states. This critical finding points to the possibility of beneficial L. iners-dominated microbiomes that had not been evidenced previously. A *L. iners* dominated microbiota is frequently observed among Black and Hispanic women (30 and 35%, respectively) [1, 11] and, among these groups, the incidence of BV is higher than White or Asian women [49, 50] and *L. iners* is associated with the prevalence and incidence of BV [41, 51]. In the vaginal microbiomes of Asian women*, L. iners* predominated communities are also frequently detected with multiple studies citing a prevalence near 40% (35%, *n* = 22 in this study, ca. 40%, *n* = 38 in France et al. [11], 42.7%, *n* = 24 in Ravel et al. [1] and 45%, *n* = 30 of women in Xu et al. [52]), and there is a lower prevalence and incidence of BV but a positive association between *L. iners* and BV has yet to be identified [49, 50, 52, 53]. MgSs and mgCST classification may provide insight that would need to be further demonstrated into the contradictory associations of *L. iners* and increased risk of BV in different racial groups. For example, of all *L. iners* mgCSTs (10–14), Asian and White women in this study were most likely to be in mgCST 12 (predominated by *L. iners* mgSs 3), while those from Black women were distributed similarly across *L. iners* mgCSTs even among samples with low Nugent scores. We hypothesize that *L. iners* mgCST 12 might represent a more stable ecological network of strains of *L. iners*. Along the same lines, multiple studies among Asian individuals do not find “*Ca.* Lachnocurva vaginae” as a prevalent member of the vaginal microbiota during BV [52, 53]. Together, these observations may have implications for the disproportionate burden of BV among racial groups. For example, selective pressures by the host environment may result in niche specialization by vaginal bacteria. Sources of selective pressure could relate to host-provided nutrient availability (e.g., mucus glycan composition), the host’s innate and adaptive immune system, the circulation of other species’ mgSs in a population, or any such combination. However, because race is a social construct influenced by many other factors including stress, socioeconomic status, and education level [54, 55], it is difficult to interpret “racial” differences in the vaginal microbiome and specific hypotheses should be tested to better understand the findings reported here.

Our analyses also identified a specific set of *L. iners* genes associated with positive Amsel-BV diagnoses. Macklaim et al. 2018 reported marked differences in *L. iners* gene expression between two control patients versus two diagnosed with BV, including increased CRISPR-associated proteins gene expression in BV samples [56]. However, our mgSs analysis of *L. iners* indicates that it is not simply alterations in gene expression of a common gene pool that differentiates BV from non-BV microbiomes, but *L. iners* mgSs that also differ. Microbiomes from women with Amsel-BV diagnoses were enriched for host immune response evasion and host-colonization functions by *L. iners*. For example, serine/threonine-protein kinases (STPKs) contribute to resistance from phagocytosis by macrophage, invasion of host cells including epithelia and keratinocytes, antibiotic resistance, disruption of the NF-κB signaling pathway, and mucin binding [57]. Bacteria attached to host cells (clue cells) is a hallmark of high Nugent scores (a bacterial morphology-based definition of bacterial vaginosis) and a criterion in Amsel-BV diagnoses [21, 25]. *L. iners* can appear as Gram-variable cocci (like *G. vaginalis*) or rods [46, 58], and our data suggest that certain strains of *L. iners* (specifically those containing gene cluster 6) may adhere to epithelial cells, contributing to the appearance of clue cells. In addition, epithelial cell adherence could make certain *L. iners* strains more difficult to displace in the vaginal environment and contribute to the common observation of *L. iners* following antibiotic treatment [59].

Several distinct mgCSTs are associated strongly with Amsel-BV. Critically, these data support the need for an improved definition of BV and the importance of a personalized approach to treatment. “*Ca.* Lachnocurva vaginae” predominated mgCSTs were strongly associated with asymptomatic Amsel-BV and contained more high Nugent scores than other mgCSTs. Conversely, intermediate Nugent scores were most prevalent in *Gardnerella*-predominated mgCSTs, and only three of these six mgCSTs were associated with Amsel-BV, which suggests that not all *Gardnerella-*dominated microbiomes are related to Amsel-BV. *Gardnerella* contains vast genomic diversity, supporting a split into different genomospecies [40, 60, 61]. Because different genomospecies can co-exist our data show that *Gardnerella* predominated mgSs represent unique combinations of genomospecies and strains of these genomospecies. MgCSTs 20-22 contain high *Gardnerella* genomospecies diversity and were associated with positive Amsel-BV diagnoses in studies using qPCR or transcriptomic data to define *Gardnerella* species [31, 32, 60]. Our data corroborate these reports. We hypothesize that in mgCSTs with higher numbers of *Gardnerella* genomospecies that there are more gene variants coding for virulence factors like cholesterol-dependent pore-forming cytotoxin vaginolysin and neuraminidase sialidase present, thus expanding functional redundancy of these enzymes and potentially contributing to the association with positive Amsel-BV diagnoses [62–64]. However, mgCST 24 which is comprised of *Gardnerella* mgSs 4 (and specifically genomospecies *G. vaginalis* and *G. swidsinkii*), has relatively lower *Gardnerella* genomospecies diversity and was also highly associated with Amsel-BV and symptomatic Amsel-BV. Together these data suggest that enumeration and classification of *Gardnerella* genomospecies may prove to be an important diagnostic of different “types” of Amsel-BV which could inform treatment options. For example, it is possible that harboring more *Gardnerella* genomospecies may predict BV recurrence following metronidazole treatment, suggesting the need for a different approach to treatment. Alternatively, some *Gardnerella* genomospecies may be important and novel targets of therapy.

Antibiotic treatment is indicated for BV diagnosis by Amsel clinical criteria when 3 of 4 criteria are observed, which is estimated to occur in fewer than half of women with BV [24, 65, 66]. In research settings, both symptomatic and asymptomatic Amsel-BV can be evaluated. Indeed, in the observational research studies included in this analysis where Amsel criteria were evaluated along with whether participants reported symptoms or not, symptomatic Amsel-BV accounted for only 12% of Amsel-BV cases and 30% of these were in mgCST 24 (dominated primarily by *G. swidsinkii* and *G. vaginalis*). We hypothesize that the high prevalence of BV recurrence post-treatment may be due to the heterogeneity in the genetic makeup of the microbiota associated with BV as revealed by mgCSTs. MgCSTs reduce this heterogeneity resulting in more precise estimates of risk. Furthermore, these findings highlight the potential importance of developing specialized treatments that target “types” of BV.

The mgCST framework can also be used to identify vaginal microbiomes that are associated with positive health outcomes. For example, mgCSTs are predominated by different *L. crispatus* mgSs varied in their association with low Nugent scores, the number of *L. crispatus* strains present, and the longitudinal stability of communities. The vaginal microbiome can be dynamic [67–69]. Shifts from *Lactobacillus* to non-*Lactobacillus* predominated microbiota can increase the risk of infection following exposure to a pathogen. Our study identified *L. crispatus* mgCSTs with variable stability, suggesting that not all *L. crispatus* predominated microbiomes are functionally similar and may be differently permissive to infection. Those found to be associated with higher stability may reduce the window of opportunity for pathogens to invade. Microbiome stability may be related to both the diversity of other non-*Lactobacillus* members of the microbiome and/or the number of *L. crispatus* strains present. In any case, our study shows that there is a range of protective abilities even among *L. crispatus* predominated communities. This information could be critical in selecting and assembling strains of *L. crispatus* to design novel live biotherapeutics products aimed to restore an optimal vaginal microenvironment.

Data are still emerging about what factors contribute to vaginal strain assemblages and what rules define their biology and ecology. However, such assemblages can now be detected and further characterized using the concepts of mgSs and mgCSTs presented here. The use of metagenomic sequencing and mgSs and mgCSTs will contribute to a much-needed functional understanding of the role of the vaginal microbiome in reproductive health outcomes. Our findings support the hypothesis that genetic and functional differences between vaginal microbiomes, including those that may look compositionally similar, are critical considerations in vaginal health [7]. To aid in further exploration, we also provide a validated classifier for both mgSs and mgCSTs at https://github.com/ravel-lab/mgCST-classifier/blob/main/README.md. The classifiers are dependent upon VIRGO and therefore rely on VIRGO-based taxonomic and gene function annotations. Additionally, as noted in the validation of external studies, some mgCSTs may be cohort-specific and thus may not be generalizable for all studies.

## Conclusion

MgCSTs reveal differences between vaginal microbiomes both compositionally and functionally, and thus more finely describe the vaginal microbiome. Associations between mgCSTs and bacterial vaginosis highlight the multi-faceted aspects of the condition and call for new and expanded definitions. Furthermore, mgCSTs have the potential to predict clinical outcomes such as recurrent BV and develop specialized treatments that target different types of BV. Further, we provide tools for the classification of mgSs and mgCST that have the potential for use and harmonization of analytical strategies in future studies.

## Methods

### Study cohorts

Raw metagenomic data from 1890 vaginal samples were used in this study (Supplementary file S6). This included publicly available metagenomes including those used in the construction of the vaginal non-redundant gene database, VIRGO (virgo.igs.umaryland.edu, *n* = 342) [29], the University of Maryland Baltimore Human Microbiome Project (UMB-HMP, *n* = 677, PRJNA208535, PRJNA575586, PRJNA797778), the National Institutes of Health Human Microbiome Project (NIH-HMP, *n* = 174, phs000228 [70]), metagenomes from Li et al. [71] (*n* = 44, PRJEB24147), the Longitudinal Study of Vaginal Flora and Incident STI (LSVF, *n* = 653, dbGaP project phs002367). All samples in LSVF (*n* = 653) and some in UMB-HMP (*n* = 20) had clinical diagnosis information about Amsel-BV. Amsel-BV was diagnosed based on the presence of 3 out of 4 Amsel’s criteria [21] and in this study, Amsel-BV was classified as symptomatic Amsel-BV when a participant self-reported complaints of vaginal discharge, irritation, itching, burning, foul odor, and other [43, 55]. At the time of these studies, gender identity information was not collected. We know all women responded to recruiting materials which included “women” or “woman”. In addition, individuals are referred to as women in previous publications, thus we refer here to individuals as “woman” or “women” to maintain consistency. Cohorts used in this study are described in Supplementary file S9.

### Sequence processing and bioinformatics

Host reads were removed from all metagenomic sequencing data using BMTagger and the GRCh38 reference genome, and reads were quality filtered using trimmomatic (v0.38, sliding window size 4 bp, Q15, minimum read length:75 bp) [72]. Metagenomic sequence reads were mapped to VIRGO using bowtie (v1; parameters: -p 16 -l 25 --fullref --chunkmbs 512 --best --strata -m 20 --suppress 2,4,5,6,7,8), producing a taxonomic and gene annotation for each read. Samples with fewer than 100,000 mapped reads were removed from the analysis (*n* = 59). The number of reads mapped to a gene was multiplied by the read length (150 bp) and divided by the gene length to produce a coverage value for each gene. Conserved domain and motif searches were performed with CD-SEARCH and the Conserved Domain Database (CDD), using an e-value threshold of 10^−4^. The taxonomic composition table generated using VIRGO was run through the vaginal CST classifier VALENCIA [11].

### Metagenomic subspecies

For each species, a presence/absence matrix was constructed from a metagenome which included all genes with at least 0.5× average coverage after normalizing for gene length. Metagenomic subspecies were generated for species present (> 75% estimated median number of genes encoded in reference genomes from the Genome Taxonomy Database [73], see Table S3) in >20 samples using binary gene counts and hierarchical clustering with Ward linkage of sample Jaccard distances calculated using the vegdist function from the vegan package (v2.5-5) [74] in R (v. 3.5.2). MgSs were defined using the dynamic hybrid tree cut method (v.1.62-1) and minClusterSize = 2 [75]. Heatmaps of gene presence/absence were constructed for each species using the gplots package heatmap.2 function [76] (Supplementary file S5). MgSs were tested for associations with low species coverage using logistic regression in which the mgSs was the binary outcome and the log_10_-transformed coverage of the species was the predictor. Tests were done at the participant level; if a participant had more than one sample and both samples were the same mgSs, only one sample was used, but if the mgSs differed, the samples were included in each. *P*-values were adjusted for multiple comparisons using Bonferroni correction. Significant dependence was observed in multiple mgSs of *Atopobium vaginae, Gardnerella*, and *Lactobacillus iners* (Table S4). For these species, the classifiers were built using samples with ≥ 5.5e5 reads. The cluster stability of each mgSs was evaluated using the clusterboot function of the R package fpc (v 2.2-10) [77, 78] and 100 bootstraps.

### Metagenomic CSTs

Using gene abundance information (normalized by gene length and sequencing depth), we estimated the proportion of vaginal species in each sample. For species that were sub-divided into mgSs, the mgSs proportion in a sample was equal to the proportion of the species in that sample. When a species was present in a sample but with too few genes present to constitute a mgSs (< 75% estimated median number of genes encoded in reference genomes), it was labeled as “mgSs 0”. Samples in the resulting compositional table were hierarchically clustered using Jensen-Shannon distances. Clusters were defined using the dynamic hybrid tree cut method (v.1.62-1) [75]. A heatmap for metagenomic CSTs was produced using the gplots package heatmap.2 function (Fig. 1) [76]. For each mgCST, the mgSs most frequently observed (prevalence) and the mgSs with the greatest mean abundance were noted. Cluster stability was evaluated with the clusterboot function from the fpc package (v. 2.2-10) using ward linkage of Jensen-Shannon distances and 100 bootstraps [77]. Cluster stability ≥ 0.75 is considered high stability.

#### Statistical analysis of the association between mgCST and age, race, Nugent score, vaginal pH, and BV

For those samples with race, age category (15–20, 21–25, 56–30, 31–35, 36–40, 41–45 years old), Nugent score category (0–3, 4–6, 7–10), vaginal pH category (pH < 4.5 or pH ≥ 4.5), or Amsel-BV diagnoses information (Table 1), the Cochran-Mantel-Haenszel Chi-Squared Test (CMH test, “mantelhaen.test” from the samplesizeCMH R package, v 0.0.0, github.com/pegeler/samplesizeCMH) was used to determine associations with mgCSTs while accounting for source study (the confounding variable). The CMH test evaluates associations between two binary variables (i.e., “mgCST X or not” and “high Nugent score or not”). Tests were done at the participant level; if a participant had more than one sample and both samples were the same mgCST, only one sample was used, but if the mgCSTs differed, the samples were included in each.

### Statistical analysis of the association between mgSs, gene clusters, and BV

For both *L. iners* and *Gardnerella*, associations between mgSs and Amsel-BV were evaluated at the participant level using chi-square analyses which compared the proportion of Amsel-BV positive to negative participants within mgSs to those in all participants containing any *L. iners*. For both *L. iners* and *Gardnerella*, associations between gene clusters and Amsel-BV were evaluated at the participant level using chi-square analyses which compared the proportion of Amsel-BV positive to negative participants within a gene cluster to those in all participants containing any *L. iners* or *Gardnerella* gene cluster, respectively. Gene cluster presence in a sample was defined as the presence of ≥ 30% of genes in a gene cluster.

### Longitudinal stability and Shannon diversity of L. crispatus mgSs

For participants in the HMP cohort that contributed multiple samples with at least one sample assigned to an *L. crispatus* mgSs, the Yue-Clayton θ was measured to define microbiota stability for each participant [79]. Here, 16S rRNA gene amplicon sequencing-based CSTs from all samples from a participant [68] were used to produce a reference centroid, and then each sample was compared to that reference (Yue-Clayton’s θ). The mean θ for each participant represented the overall microbiota compositional stability. Values closer to 1 indicate high compositional stability. The number of strains in each sample was compared between mgSs using the Wilcoxon signed rank test. Shannon’s *H* diversity index was calculated for each sample using the vegan package diversity function. Shannon Diversity was compared between mgSs using the Wilcoxon signed rank test.

### Estimating the number of L. crispatus strains

The number of *L. crispatus* strains in a mgSs was estimated using a pangenome accumulation curve which was generated by mapping the gene contents of publicly available isolate genome sequences (Table S3) to VIRGO (blastn, threshold: 90% identity, 70% coverage). Bootstrap (*n* = 100) combinations of N (*N* = 1 to 61) isolates were selected and the number of unique *L. crispatus* Vaginal Orthologous Groups (VOGs; provided in the VIRGO output [29]) encoded in their genomes was determined. An exponential curve relating the number of isolates to the number of VOGs detected was then fit to the resulting data and produced the equation: Y = 2057N^0.14^ where Y is the number of *L. crispatus* VOGs detected, and N is the estimated number of strains. This equation was then used to estimate the number of *L. crispatus* strain’s detected in each metagenome based on the observed number of *L. crispatus* VOGs in each metagenome. The number of strains in each sample was compared between mgSs using the Wilcoxon signed rank test.

### Construction of the random forests for mgSs classification

We constructed random forests for the classification of mgSs using the R package randomForestSRC v2.12.1R [80]. For mgSs, a random forest was built for each species (*n* = 28) where the training data contained presence/absence values of genes. Gene presence was defined as above for mgSs. We implemented random forest classification analysis with all predictors included in a single model. For each mgSs random forest, predictors were all genes in a species. Ten-fold cross-validation (90% of data as training, 10% as testing) was performed wherein each training set was used to build and tune a random forest model using tune “tune.rfsrc”. A random forest model using optimal parameters was then used to predict mgSs classifications for the test set and out-of-bag error estimates (misclassification error) were reported. The overall misclassification error is the average misclassification error from each fold and the “correct” assignment is based on the original hierarchical clustering assignment. The final models included all data and the optimal tuning parameters determined for that species. For mgSs assignment, the mgSs which provide the highest probability (based on the proportion of votes in the tree) is used for assignment. The user is provided with both the assignment, as well as the probability of that assignment as a measure of confidence.

### Construction of the nearest centroid classifier for mgCSTs

Using mgCSTs as defined above, reference centroids were produced using the mean relative abundances of each mgSs in a mgCST. For classification, the similarity of a sample to the reference centroids is determined using Yue-Clayton’s θ [79]. Compared to Jensen-Shannon, the Yue-Clayton *θ* measure depends more on the high relative abundance metagenomic subspecies than those at lower relative abundances. Samples are assigned to the mgCST to which they bear the highest similarity and the degree of similarity to that mgCST can be taken as a measure of confidence in the assignment. Ten-fold cross-validation was applied wherein each training set was used to build “reference” centroids and each test set was used for assignment. The misclassification error was determined by subtracting the number of correct assignments (based on the original hierarchical clustering assignment) divided by the total number of assignments from 1. The overall misclassification error is the average of misclassification error from each fold.

### Running the mgCST classifier

The required inputs are direct outputs from VIRGO [29] and include the taxonomic abundance table (“summary.Abundance.txt”) and gene abundance table (“summary.NR.abundance.txt”). It is *imperative* that taxonomic and gene column headings match those output by VIRGO. The expected output is a count table with samples as rows, taxa as columns, and counts normalized by gene length as values. Additional columns indicate the sample mgCST classification and the Yue-Clayton similarity score for all 27 mgCSTs. A heatmap is also produced showing taxon relative abundances in samples, where samples are labeled with assigned mgCSTs substantial differences may indicate either an incongruence in taxonomic or gene names or the need for an additional mgCST. The classifier is contained in an R script, which is available at https://github.com/ravel-lab/mgCST-classifier. MgSs and mgCST classifications were robust at sampling depths greater than 100,000 reads per sample (Supplementary file S10).

### Validation of the mgCST classifier in external datasets

Three external, publicly available vaginal metagenome datasets were used to validate the generalizability of mgCST assignments beyond the training dataset**.** ENA PRJEB34536 [81], NIH PRJNA576566 [82], and NIH PRJNA779415 [83]. Briefly, host reads were removed using BMTagger [84] and the GRch38_p12 human reference genome. Ribosomal RNA reads were removed using sortmerna [85] and reads were quality filtered using fastp [86] with a minimum length of 50 bp, and a mean quality of 20 in a sliding window of 4 bp. The remaining reads were processed through VIRGO using default settings (https://virgo.igs.umaryland.edu) [29] and summary tables were used to assign mgSs and mgCSTs with the mgCST Classifier. The generalizability of the mgSs and mgCST reference datasets is illustrated using the probability of assignment by the mgSs random forest classifier and the Yue-Clayton similarity scores of assigned mgCSTs.

All bioinformatic and statistical analyses are available in R Markdown notebooks (Supplementary file S7 and file S8).

### Supplementary Information

**Additional file 1:** **Figure S1.** Metagenomic CST assignments correspond to marker gene-based CST assignments primarily through the predominant taxon. However, dominance by an mgSs is not captured through marker-based CSTs. **Figure S2.** A) For some species, mgSs assignment was significantly associated with sequencing depth of a sample (see Table S4). B) When only samples with ≥ 5.5e5 reads are used in mgSs classifier random forest tree construction, mgSs assignment is no longer significantly associated with depth of sequencing. C) For each species, 10-fold cross-validation yielded random forest misclassification error estimates. **Figure S3.** A) MgSs assignment using hierarchical clustering (y-axis) are highly concordant (kappa> 0.8) with those assigned by the random forest classifier (x-axis) for that species. B) MgCST assignment using hierarchical clustering (x-axis) are highly concordant (kappa= 0.78) with those assigned by the nearest centroid classifier (y-axis). **Figure S4.** Confusion matrix and classification error estimates from 10-fold cross-validation of a nearest-centroid classifier for mgCSTs. **Figure S5.** Gene presence heatmaps for all species for which mgSs were constructed. Samples with >75% estimated median number of genes encoded in reference genomes from the Genome Taxonomy Database [63], see Table S3) were used to build mgSs. In each heatmap, samples are in the columns and genes are in the rows. Assigned mgSs are indicated in the column side colors at the top. Gene clusters are colored for each gene on the y-axis. The bottom x-axis indicates Amsel-BV diagnoses, if clinical evaluation data were available for the sample. Dendrograms were built using Ward linkage of Jaccard distances.** Figure S10.** Metagenomic subspecies (a, mgSs) and metagenomic community state types (b, mgCSTs) may be impacted with sampling (sequencing) depth. A minimum of 1 x 106 reads per sample is recommended for mgCST assignment.

**Additional file 2:** **Supplementary file S6.** Metadata and source location for all metagenomes in this study. For Amsel-BV: clinBV.asymp=Clinical diagnosis of Amsel-BV, no symptoms reported by patient; noBV=No diagnosis of Amsel-BV after evaluation; clinBV.symp=Clinical diagnosis of Amsel-BV, symptoms reported by patient; NA=No clinical evaluation.

**Additional file 3:** **Supplementary file S7.** Bioinformatic is available in R Markdown notebooks.

**Additional file 4:** **Supplementary file S8.** Statistical analyses are available in R Markdown notebooks.

**Additional file 5:** **Supplementary file S9.** Inclusion and exclusion criteria for source data cohort studies.

**Additional file 6:** **Table S1.** Metagenomic subspecies (mgSs) of the vaginal microbiome.

**Additional file 7:** **Table S2.** Associations between race, age, Nugent score, and Amsel-BV and metagenomic community state types (mgCSTs). Mantel-Haen (MH) or Chi-Squared (Chi-sq) tests evaluated significant deviations from study-wide proportions.

**Additional file 8:** **Table S3.** To estimate the number of *L. crispatus* strains in each mgSs, a pangenome accumulation curve was generated by mapping the gene contents of publicly available *L. crispatus* isolate genome sequences to VIRGO to acquired the number VOGs present in the genome.

**Additional file 9:** **Table S4.** The mgSs assignment of a sample was dependent upon log10-transformed sequencing coverage in some mgSs (adjusted *p* < 0.05). For species with multiple significant mgSs (bold), only samples with ≥ 5.5e5 reads were used to train the mgSs classifer. Dependence was tested for using a logistic regression where the response was the mgSs (binary) and the predictor was log10-transformed sequencing coverage. Testing was performed at the participant level. *P*-values were adjusted using Bonferroni correction.

## Data Availability

The classifier and reference datasets are accessible at https://github.com/ravel-lab/mgCST-classifier. Code and data used in analyses of this paper are available via https://github.com/ravel-lab/mgCST-classifier/tree/main/manuscript-files. A preprint of an older version of the manuscript is available on BioRxiv (https://doi.org/10.1101/2023.03.24.533147).
