# Correction: Integrating compositional and functional content to describe vaginal microbiomes in health and disease

**DOI:** 10.1186/s40168-024-01765-5

**Published:** 2024-02-06

**Authors:** Johanna B. Holm, Michael T. France, Pawel Gajer, Bing Ma, Rebecca M. Brotman, Michelle Shardell, Larry Forney, Jacques Ravel

**Affiliations:** 1 Institute for Genome Sciences, University of Maryland School of Medicine, Baltimore, MD USA; 2 Department of Microbiology and Immunology, University of Maryland School of Medicine, Baltimore, MD USA; 3 Department of Epidemiology and Public Health, University of Maryland School of Medicine, Baltimore, MD USA; 4 Department of Biological Sciences, University of Idaho, Moscow, ID USA

**Correction: Microbiome 11, 259 (2023)**

**https://doi.org/10.1186/s40168-023-01692-x**

Following publication of the original article [1], the author reported a descripancy between the HTML and PDF version of Fig. 1. In the PDF version there were white spaces.

The original article has been updated to correct this.
