# Supplementary material for: Integrating compositional and functional content to describe vaginal microbiomes in health and disease
Source: Microbiome. 2023 Nov 30;11:259. doi: 10.1186/s40168-023-01692-x (PMC10688475; doi:10.1186/s40168-023-01692-x)
Supplement: Supplementary file 2 — Additional file 1: Figure S1. Metagenomic CST assignments correspond to marker gene-based CST assignments primarily through the predominant taxon. However, dominance by an mgSs is not captured through marker-based CSTs. Figure S2. A) For some species, mgSs assignment was significantly associated with sequencing depth of a sample (see Table S4). B) When only samples with ≥ 5.5e5 reads are used in mgSs classifier random forest tree construction, mgSs assignment is no longer significantly associated with depth of sequencing. C) For each species, 10-fold cross-validation yielded random forest misclassification error estimates. Figure S3. A) MgSs assignment using hierarchical clustering (y-axis) are highly concordant (kappa> 0.8) with those assigned by the random forest classifier (x-axis) for that species. B) MgCST assignment using hierarchical clustering (x-axis) are highly concordant (kappa= 0.78) with those assigned by the nearest centroid classifier (y-axis). Figure S4. Confusion matrix and classification error estimates from 10-fold cross-validation of a nearest-centroid classifier for mgCSTs. Figure S5. Gene presence heatmaps for all species for which mgSs were constructed. Samples with >75% estimated median number of genes encoded in reference genomes from the Genome Taxonomy Database [63], see Table S3) were used to build mgSs. In each heatmap, samples are in the columns and genes are in the rows. Assigned mgSs are indicated in the column side colors at the top. Gene clusters are colored for each gene on the y-axis. The bottom x-axis indicates Amsel-BV diagnoses, if clinical evaluation data were available for the sample. Dendrograms were built using Ward linkage of Jaccard distances. Figure S10. Metagenomic subspecies (a, mgSs) and metagenomic community state types (b, mgCSTs) may be impacted with sampling (sequencing) depth. A minimum of 1 x 10^6^ reads per sample is recommended for mgCST assignment. [file 40168_2023_1692_MOESM1_ESM.pdf]

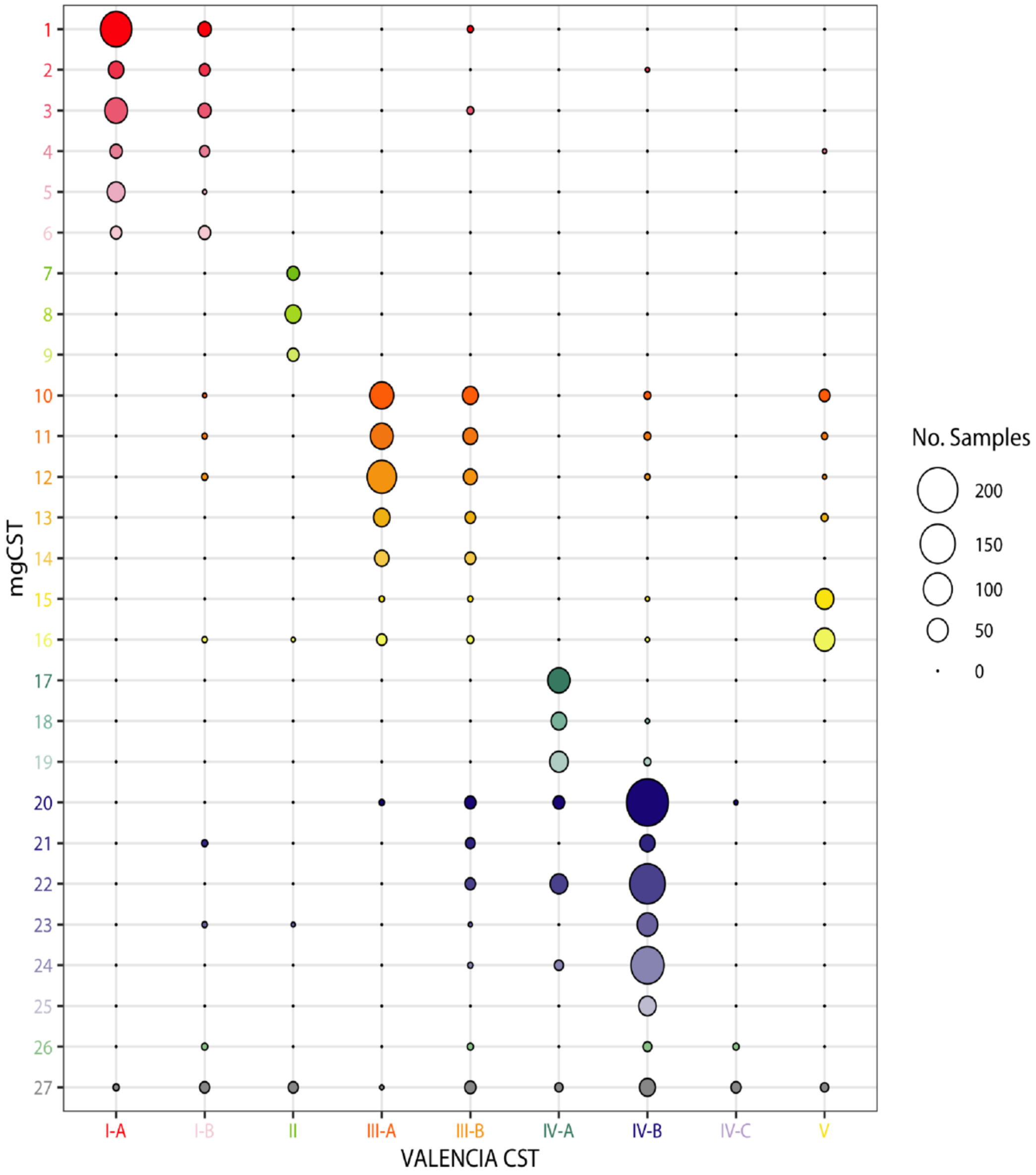

Fig. S1. Metagenomic CST assignments correspond to marker gene-based CST assignments primarily through the predominant taxon. However, dominance by an mgSs is not captured through marker-based CSTs.

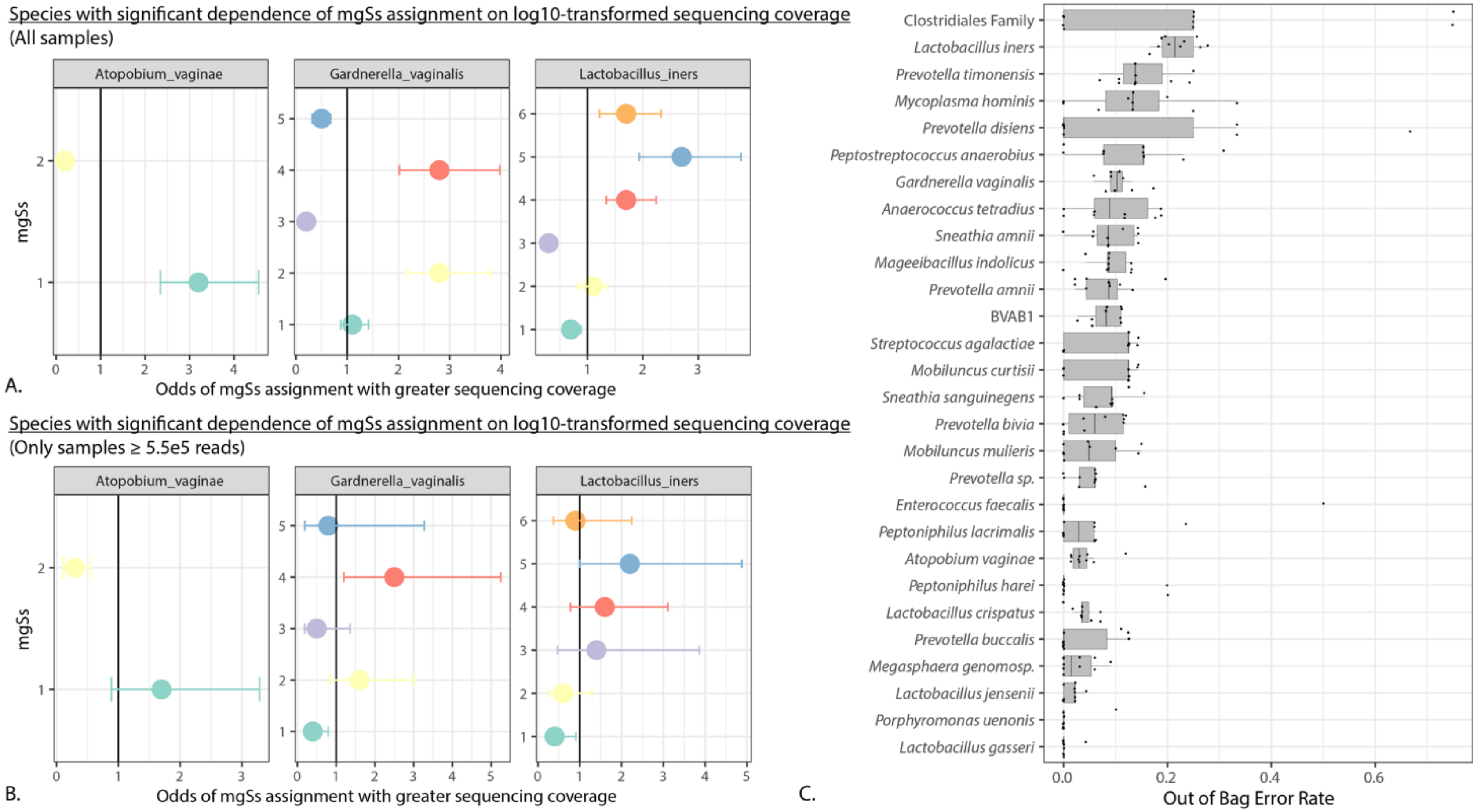

Fig. S2. A) For some species, mgSs assignment was significantly associated with sequencing depth of a sample (see Table S4). B) When only samples with ≥ 5.5e5 reads are used in mgSs classifier random forest tree construction, mgSs assignment is no longer significantly associated with depth of sequencing. C) For each species, 10-fold cross-validation yielded random forest misclassification error estimates.

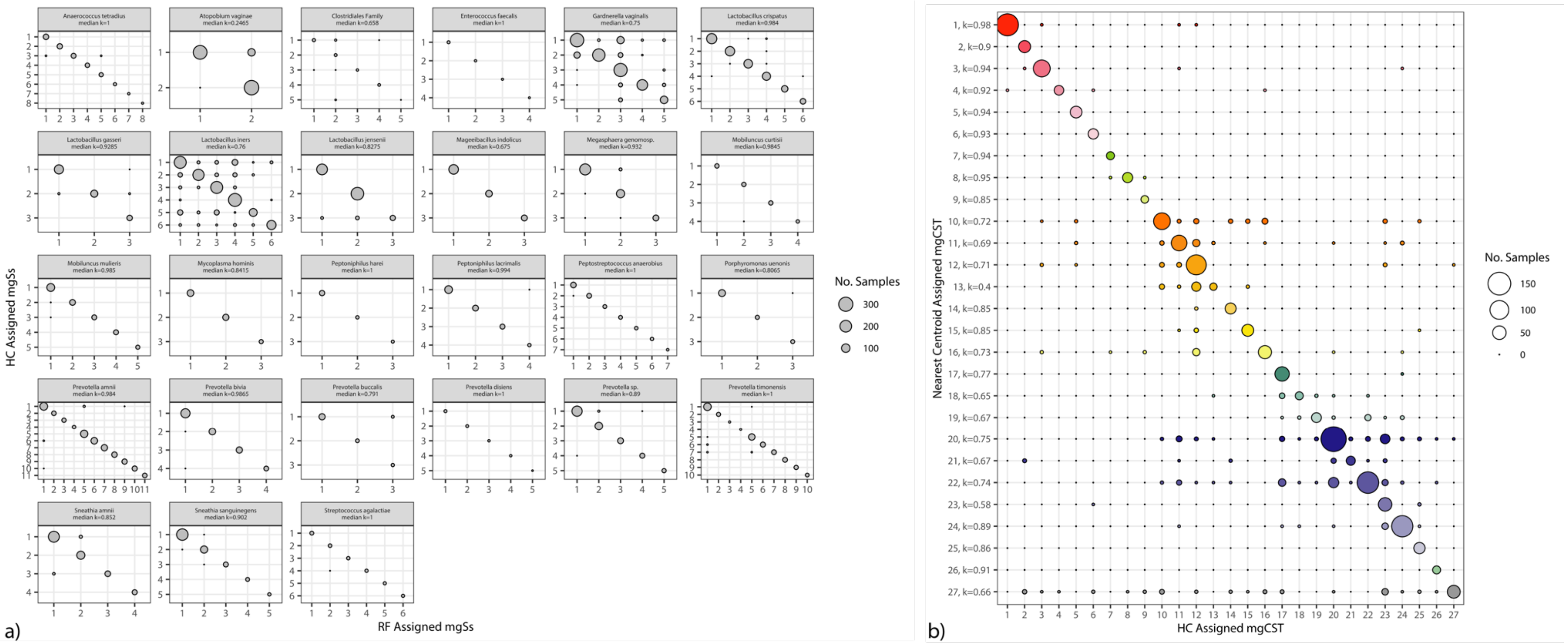

Figure S3. A) MgSs assignment using hierarchical clustering (y-axis) are highly concordant ($\boldsymbol{\kappa} > 0.8$) with those assigned by the random forest classifier (x-axis) for that species. B) MgCST assignment using hierarchical clustering (x-axis) are highly concordant ($\boldsymbol{\kappa} = 0.78$) with those assigned by the nearest centroid classifier (y-axis).

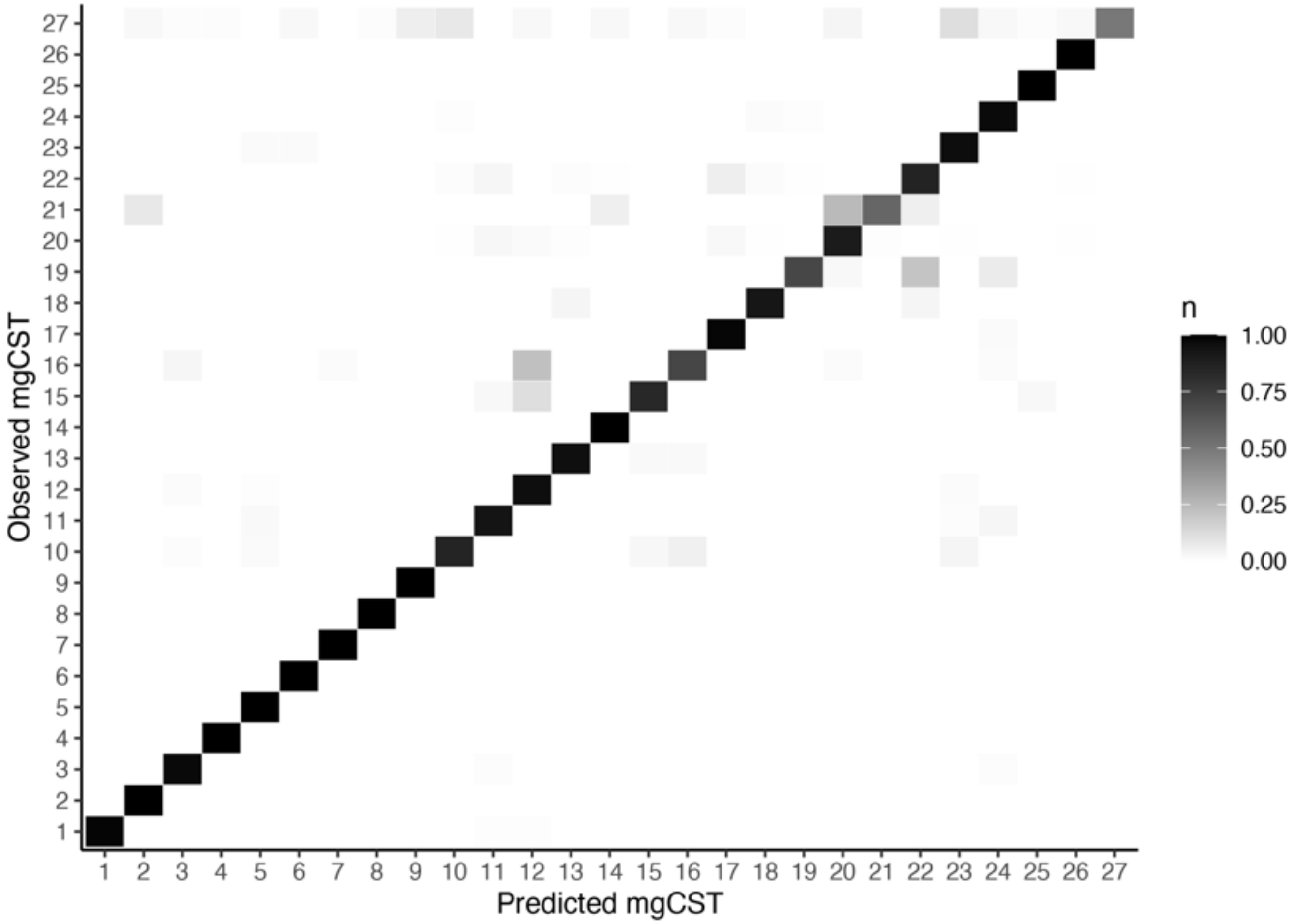

Fig. S4. Confusion matrix and classification error estimates from 10-fold cross-validation of a nearest-centroid classifier for mgCSTs.

Fig. S5. Gene presence heatmaps for all species for which mgSs were constructed. Samples with >75% estimated median number of genes encoded in reference genomes from the Genome Taxonomy Database [64], see Table S3) were used to build mgSs. In each heatmap, samples are in the columns and genes are in the rows. Assigned mgSs are indicated in the column side colors at the top. Gene clusters are colored for each gene on the y-axis. The bottom x-axis indicates Amsel-BV diagnoses, if clinical evaluation data were available for the sample. Dendrograms were built using Ward linkage of Jaccard distances.

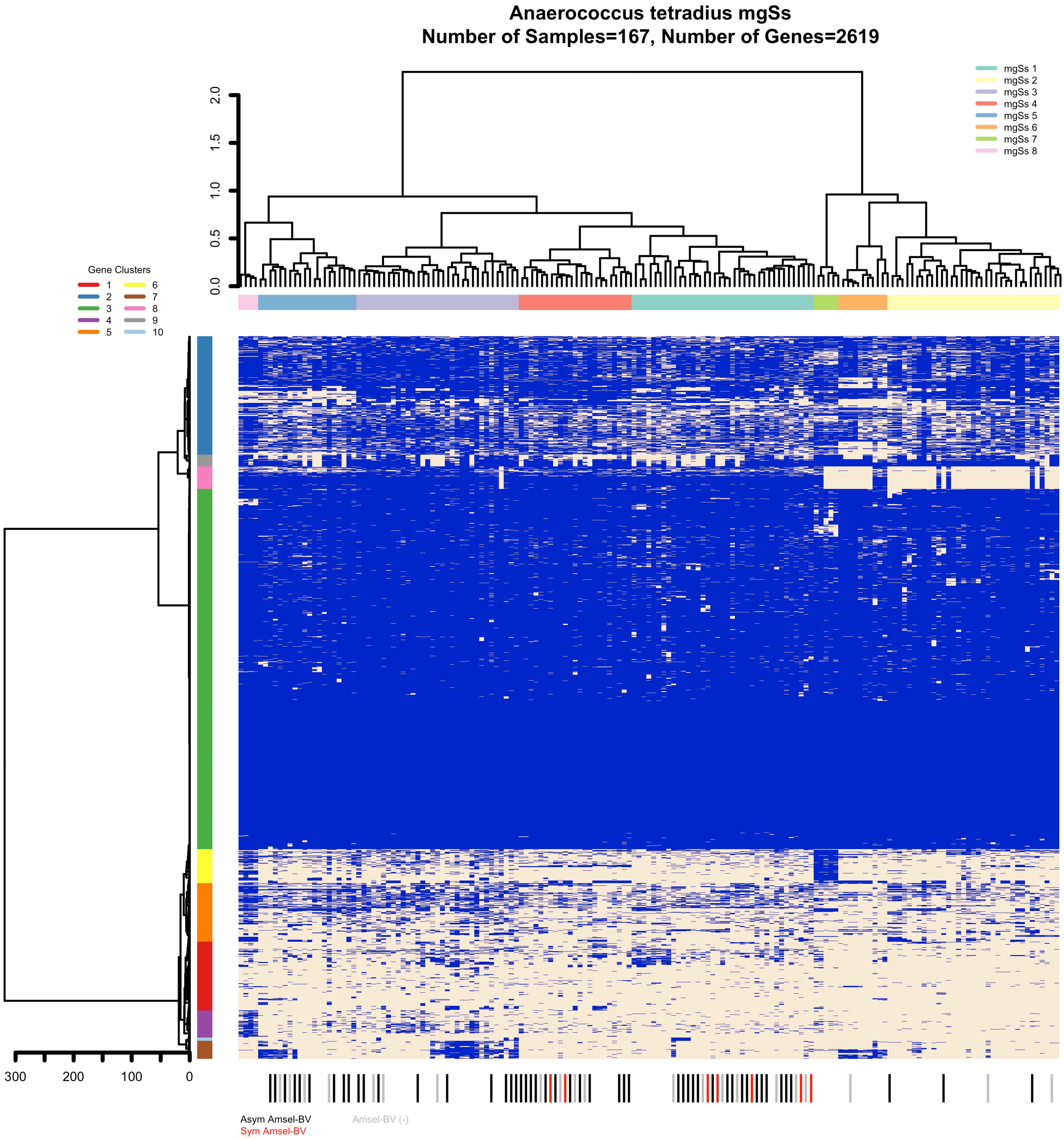

Anaerococcus tetradius mgSs
Number of Samples=167, Number of Genes=2619
mgSs 1
mgSs 2
mgSs 3
mgSs 4
mgSs 5
mgSs 6
mgSs 7
mgSs 8
0.0
0.5
1.0
1.5
2.0
Gene Clusters
1
2
3
4
5
6
7
8
9
10
300
200
100
0
Asym Amsel-BV
Sym Amsel-BV
Amsel-BV (-)

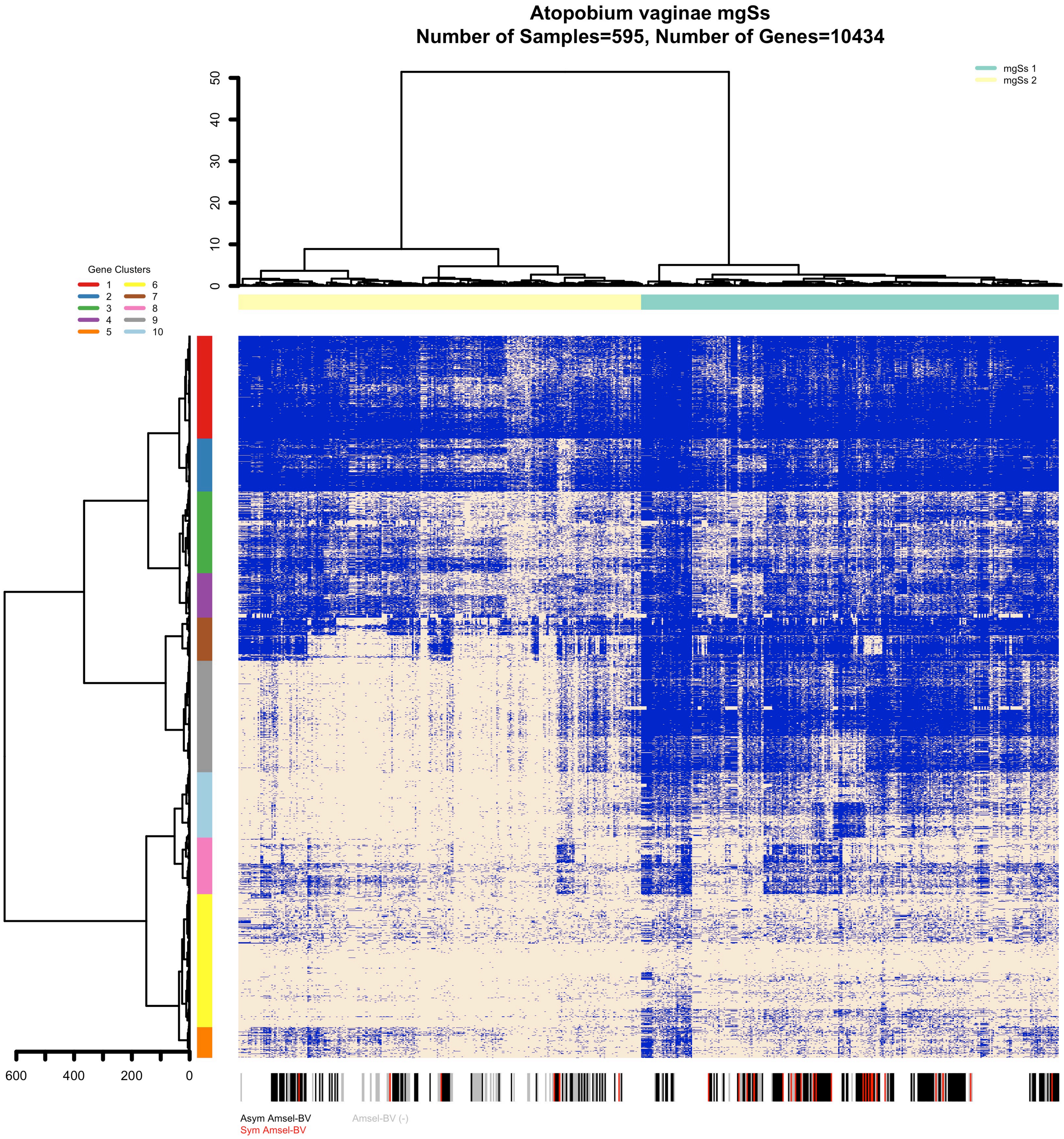
Atopobium vaginae mgSs
Number of Samples=595, Number of Genes=10434
mgSs 1
mgSs 2
50
40
30
20
10
0
Gene Clusters
1
2
3
4
5
6
7
8
9
10
600
400
200
0
Asym Amsel-BV
Sym Amsel-BV
Amsel-BV (-)

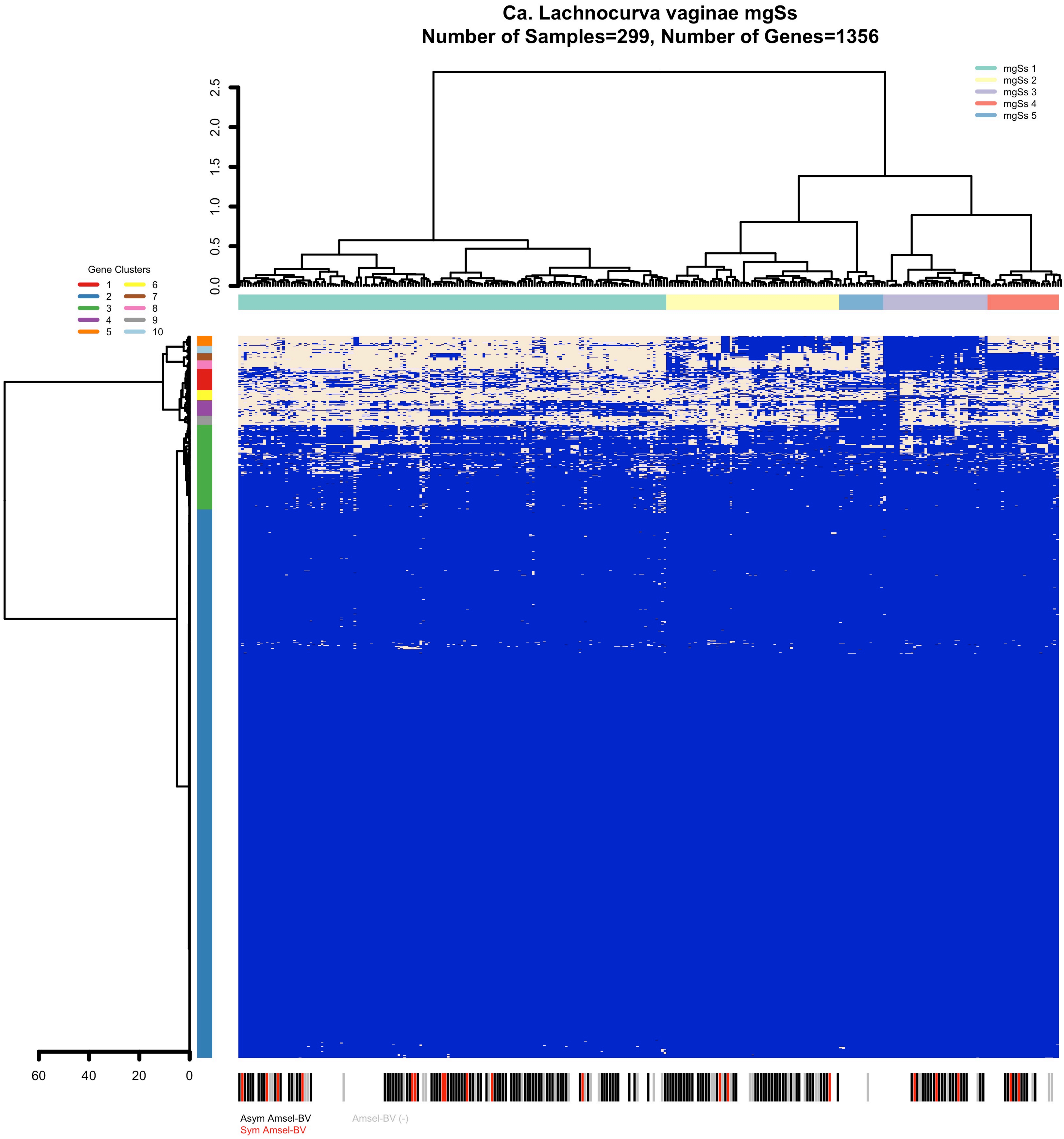
Ca. Lachnocurva vaginae mgSs
Number of Samples=299, Number of Genes=1356
mgSs 1
mgSs 2
mgSs 3
mgSs 4
mgSs 5
0.0
0.5
1.0
1.5
2.0
2.5
Gene Clusters
1
2
3
4
5
6
7
8
9
10
60
40
20
0
Asym Amsel-BV
Sym Amsel-BV
Amsel-BV (-)

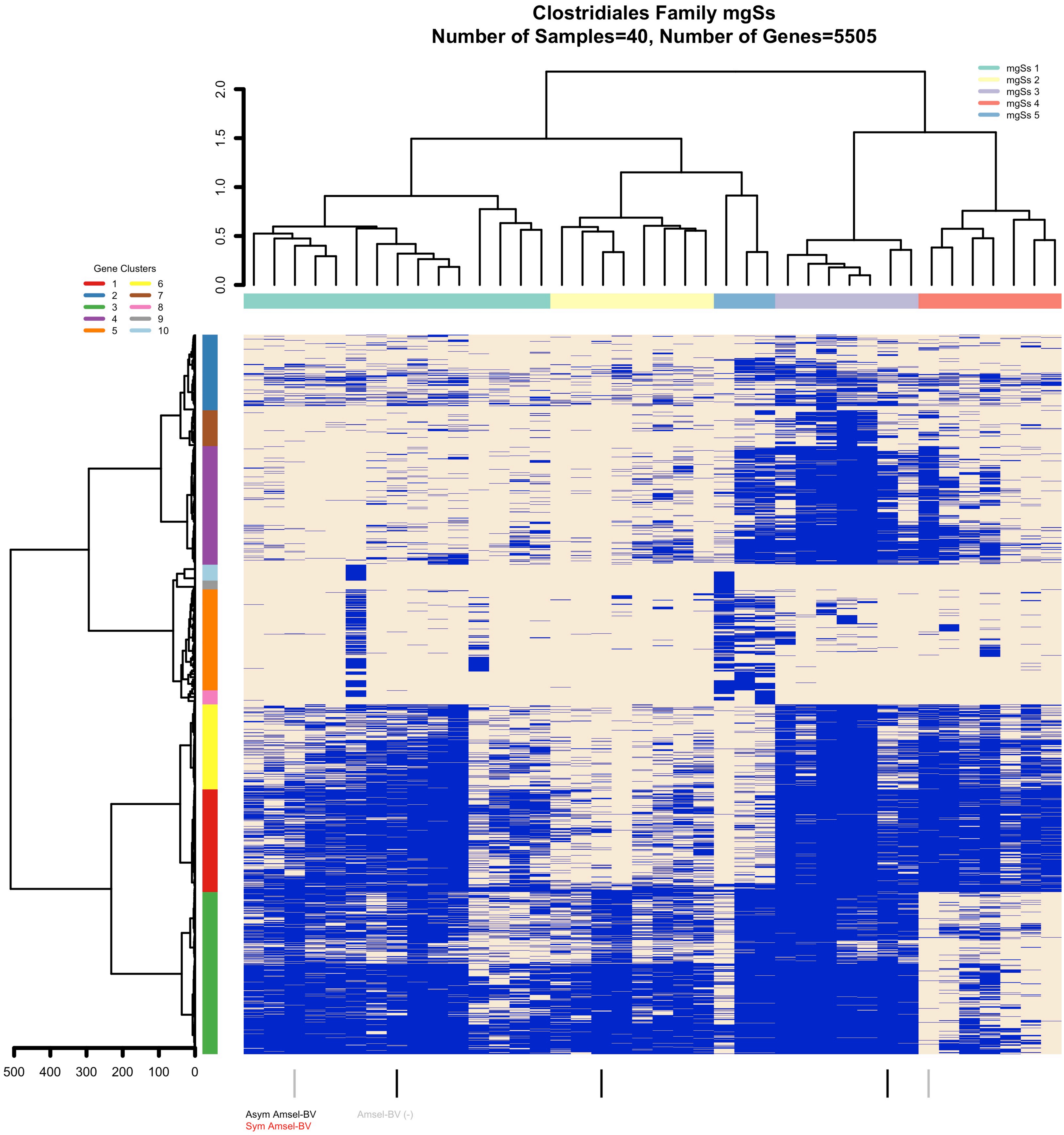
Clostridiales Family mgSs
Number of Samples=40, Number of Genes=5505
mgSs 1
mgSs 2
mgSs 3
mgSs 4
mgSs 5
0.0
0.5
1.0
1.5
2.0
Gene Clusters
1
2
3
4
5
6
7
8
9
10
500
400
300
200
100
0
Asym Amsel-BV
Sym Amsel-BV
Amsel-BV (-)

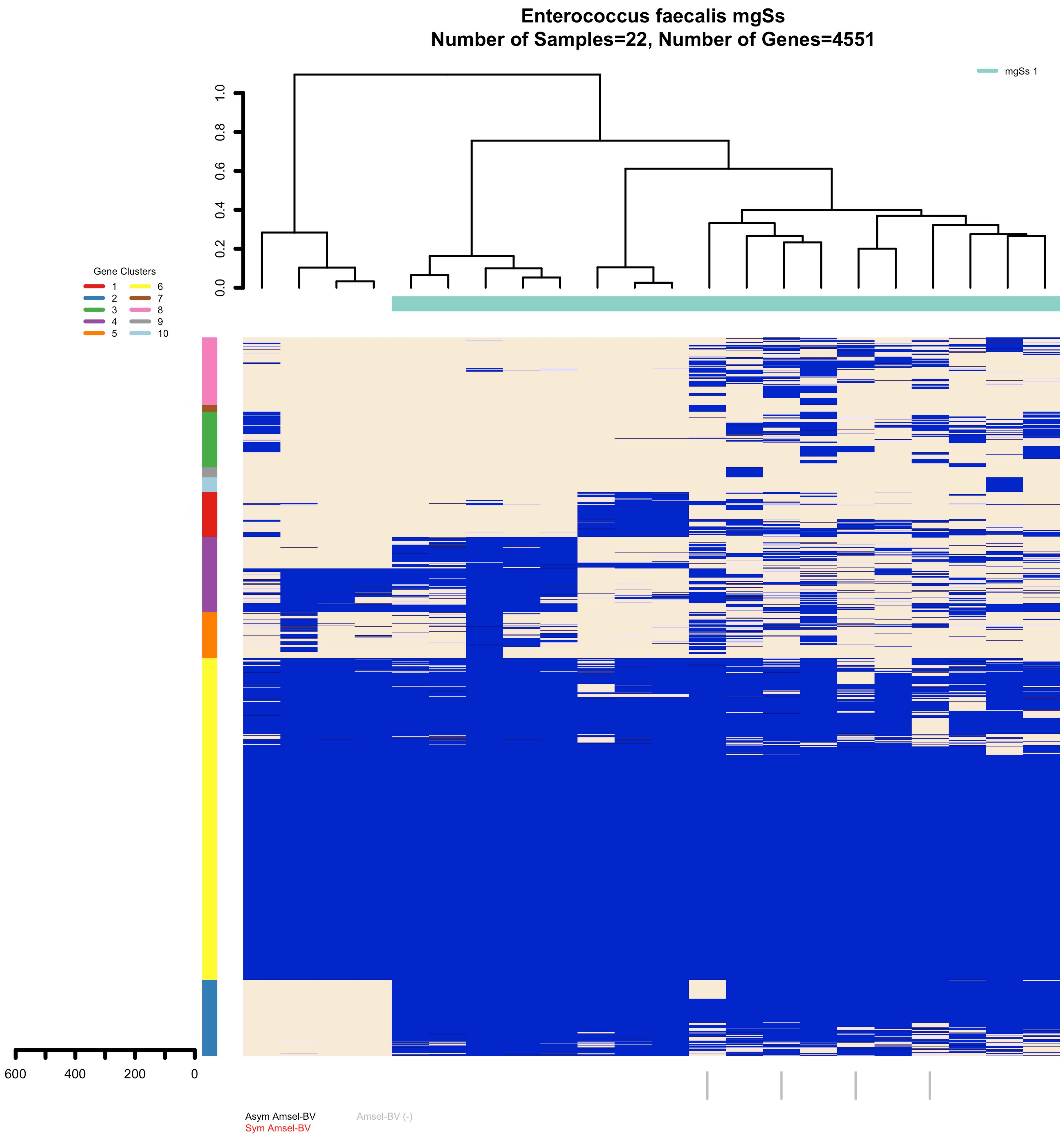

Enterococcus faecalis mgSs
Number of Samples=22, Number of Genes=4551
mgSs 1
1.0
0.8
0.6
0.4
0.2
0.0
Gene Clusters
1
2
3
4
5
6
7
8
9
10
600
400
200
0
Asym Amsel-BV
Sym Amsel-BV
Amsel-BV (-)

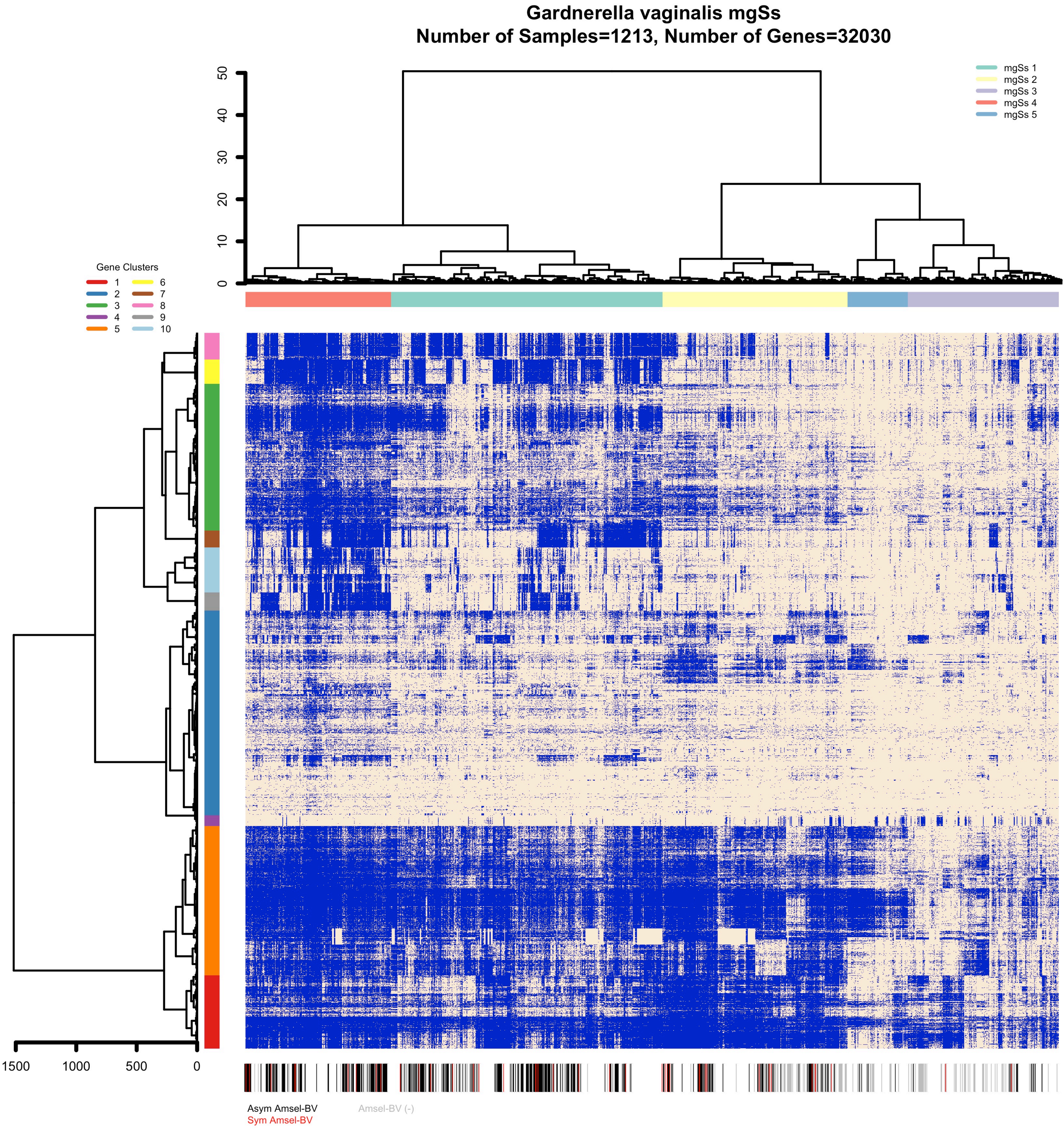
Gardnerella vaginalis mgSs
Number of Samples=1213, Number of Genes=32030
mgSs 1
mgSs 2
mgSs 3
mgSs 4
mgSs 5
Gene Clusters
1
2
3
4
5
6
7
8
9
10
0
10
20
30
40
50
1500
1000
500
0
Asym Amsel-BV
Sym Amsel-BV
Amsel-BV (-)

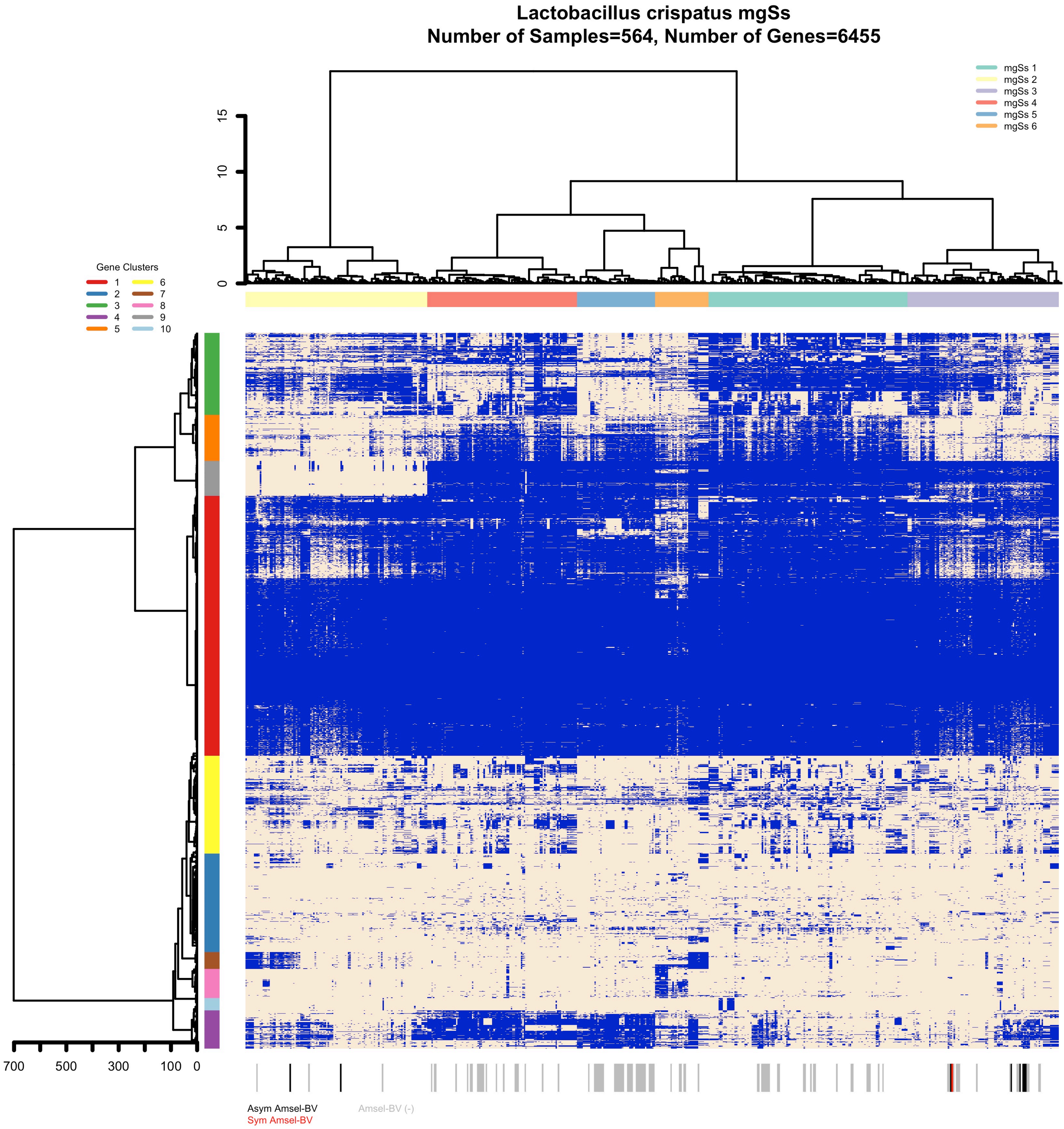

Lactobacillus crispatus mgSs
Number of Samples=564, Number of Genes=6455
mgSs 1
mgSs 2
mgSs 3
mgSs 4
mgSs 5
mgSs 6
Gene Clusters
1
2
3
4
5
6
7
8
9
10
0
5
10
15
700
500
300
100
0
Asym Amsel-BV
Sym Amsel-BV
Amsel-BV (-)

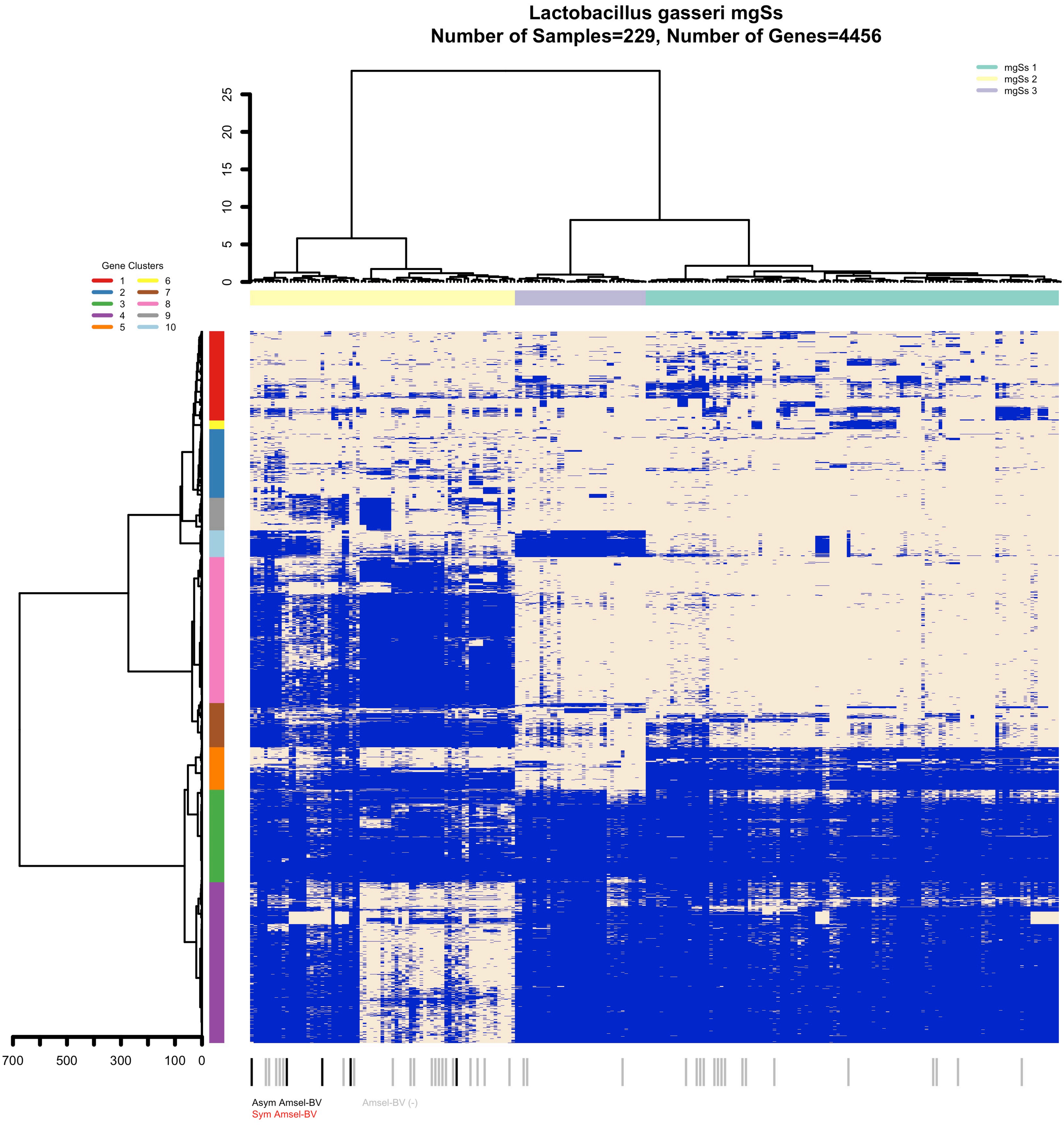

Lactobacillus gasseri mgSs
Number of Samples=229, Number of Genes=4456
mgSs 1
mgSs 2
mgSs 3
Gene Clusters
1 2 3 4 5 6 7 8 9 10
0 5 10 15 20 25
700 500 300 100 0
Asym Amsel-BV
Sym Amsel-BV
Amsel-BV (-)

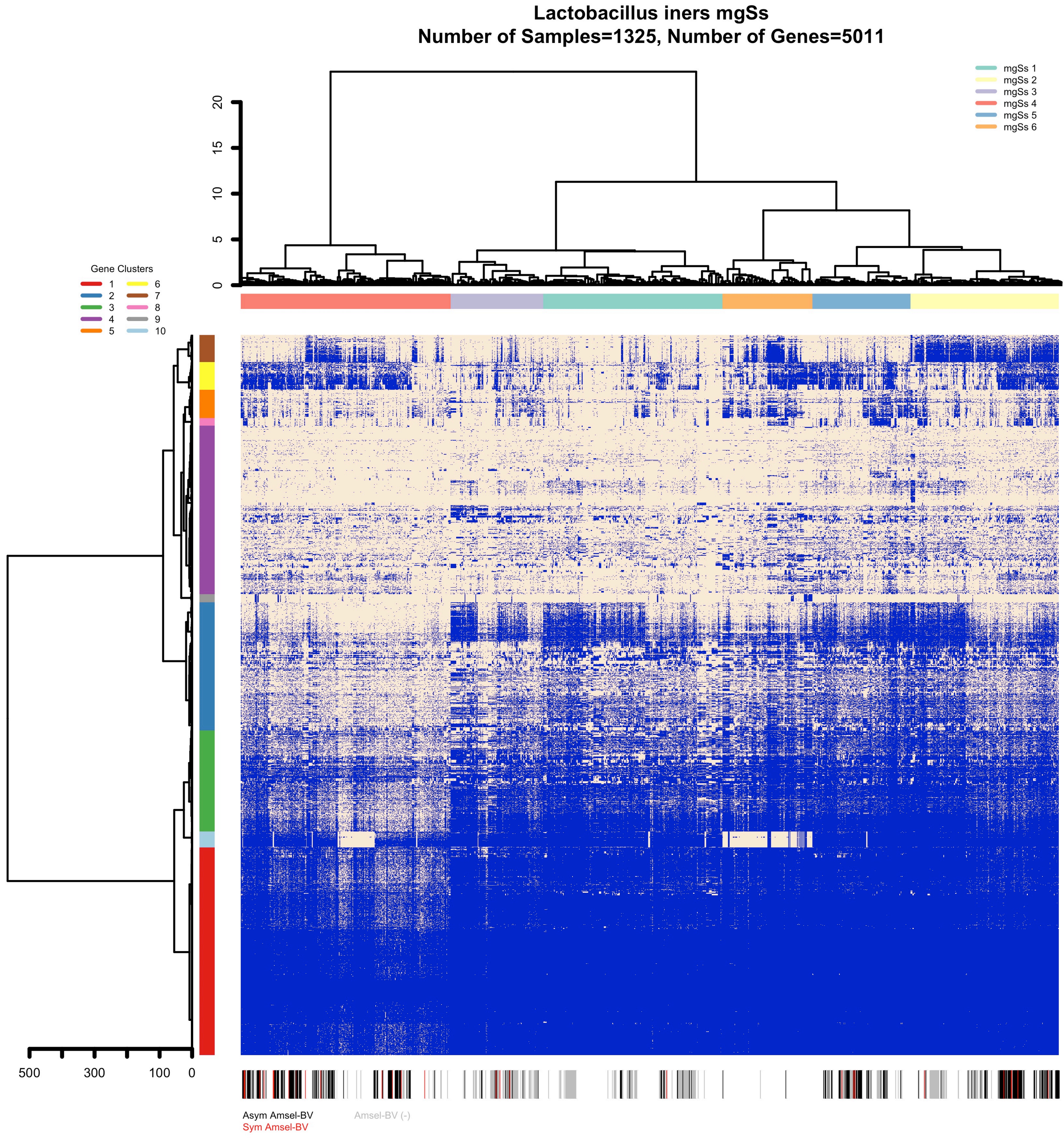

Lactobacillus iners mgSs
Number of Samples=1325, Number of Genes=5011
mgSs 1
mgSs 2
mgSs 3
mgSs 4
mgSs 5
mgSs 6
Gene Clusters
1
2
3
4
5
6
7
8
9
10
0
5
10
15
20
500
300
100
0
Asym Amsel-BV
Sym Amsel-BV
Amsel-BV (-)

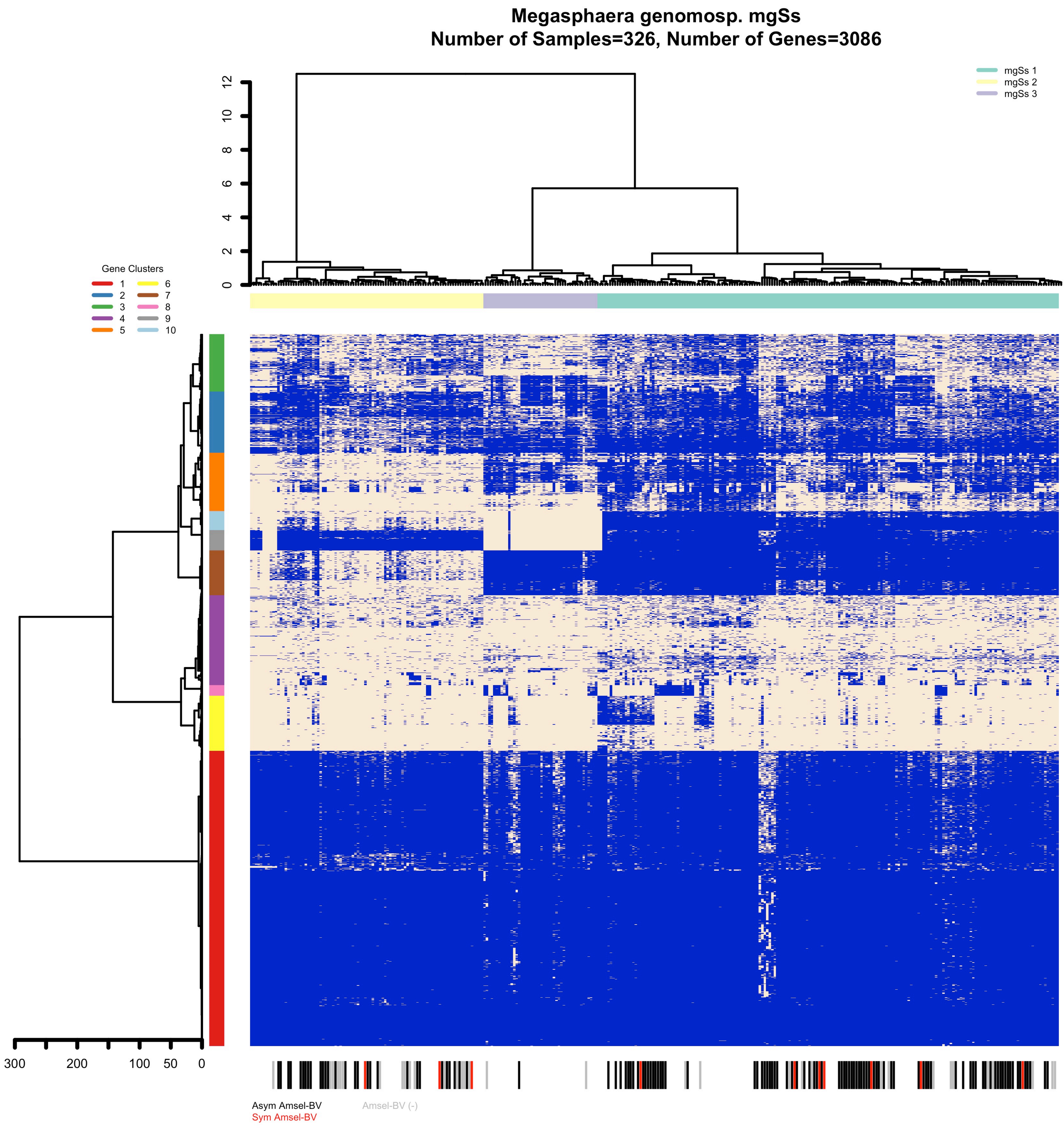
Megasphaera genomosp. mgSs
Number of Samples=326, Number of Genes=3086
mgSs 1
mgSs 2
mgSs 3
Gene Clusters
1
2
3
4
5
6
7
8
9
10
0
2
4
6
8
10
12
300
200
100
50
0
Asym Amsel-BV
Sym Amsel-BV
Amsel-BV (-)

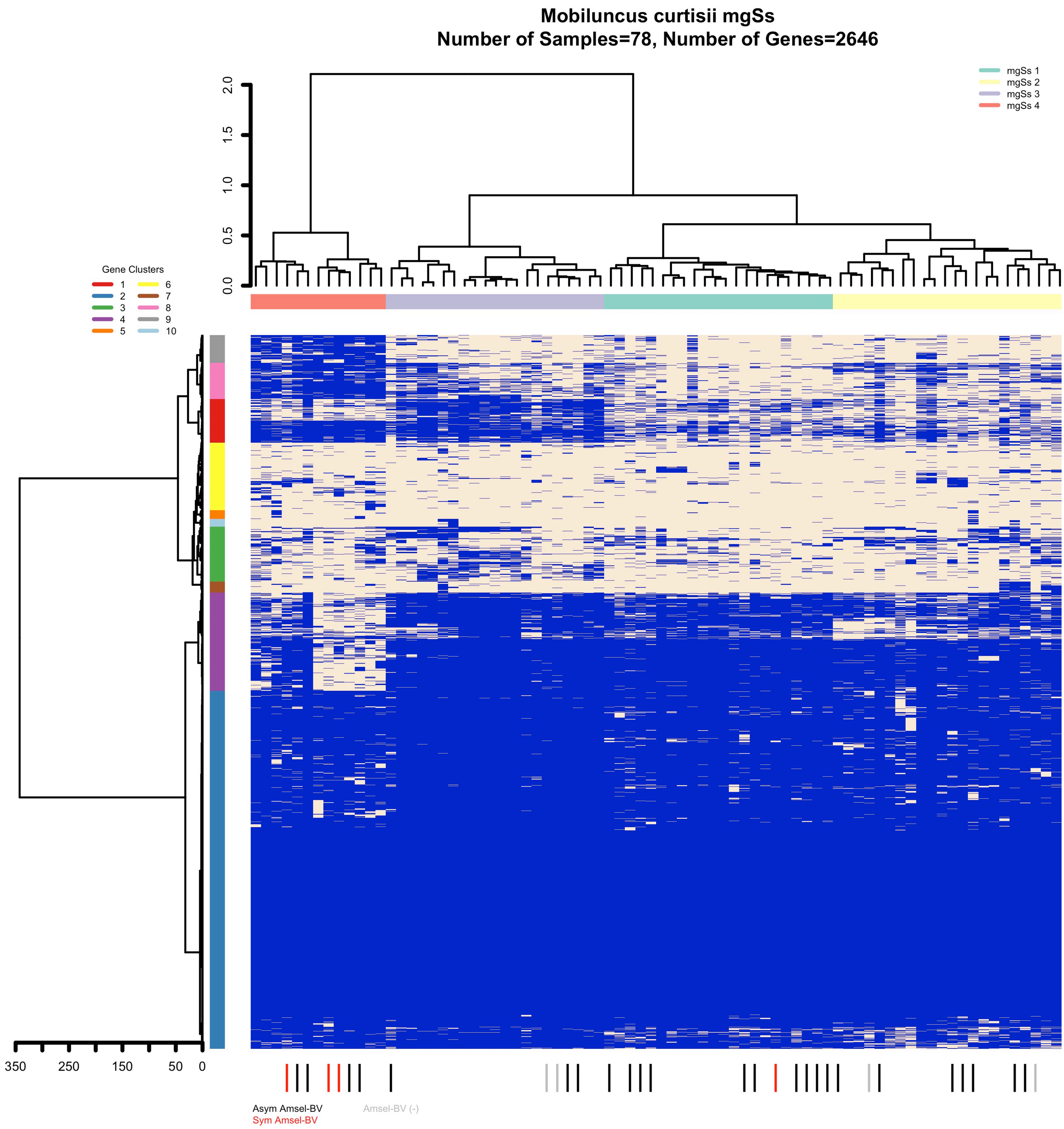

Mobiluncus curtisii mgSs
Number of Samples=78, Number of Genes=2646
mgSs 1
mgSs 2
mgSs 3
mgSs 4
Gene Clusters
1
2
3
4
5
6
7
8
9
10
0.0
0.5
1.0
1.5
2.0
350
250
150
50
0
Asym Amsel-BV
Sym Amsel-BV
Amsel-BV (-)

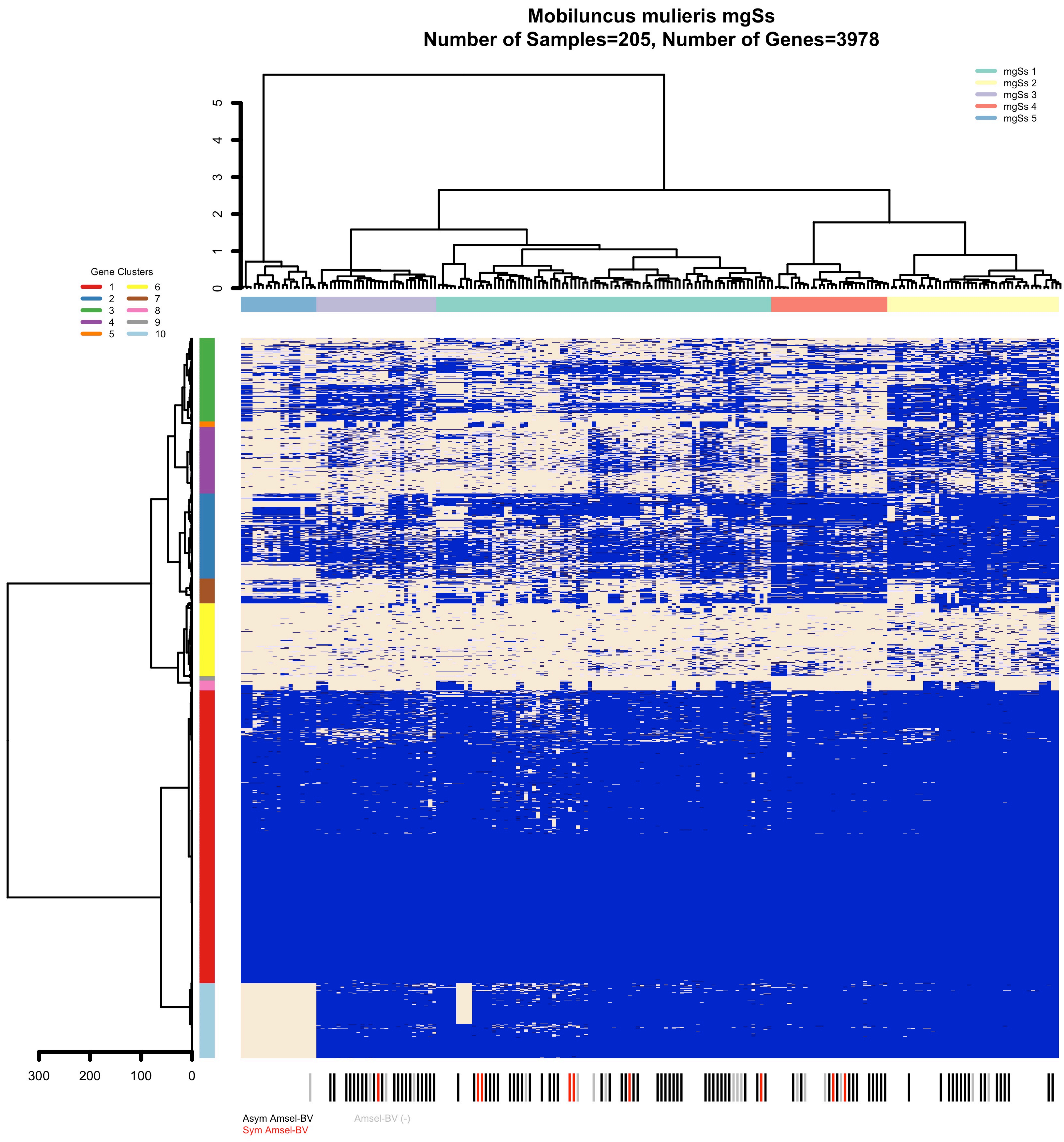

Mobiluncus mulieris mgSs
Number of Samples=205, Number of Genes=3978
mgSs 1
mgSs 2
mgSs 3
mgSs 4
mgSs 5
Gene Clusters
1
2
3
4
5
6
7
8
9
10
0
1
2
3
4
5
300
200
100
0
Asym Amsel-BV
Sym Amsel-BV
Amsel-BV (-)

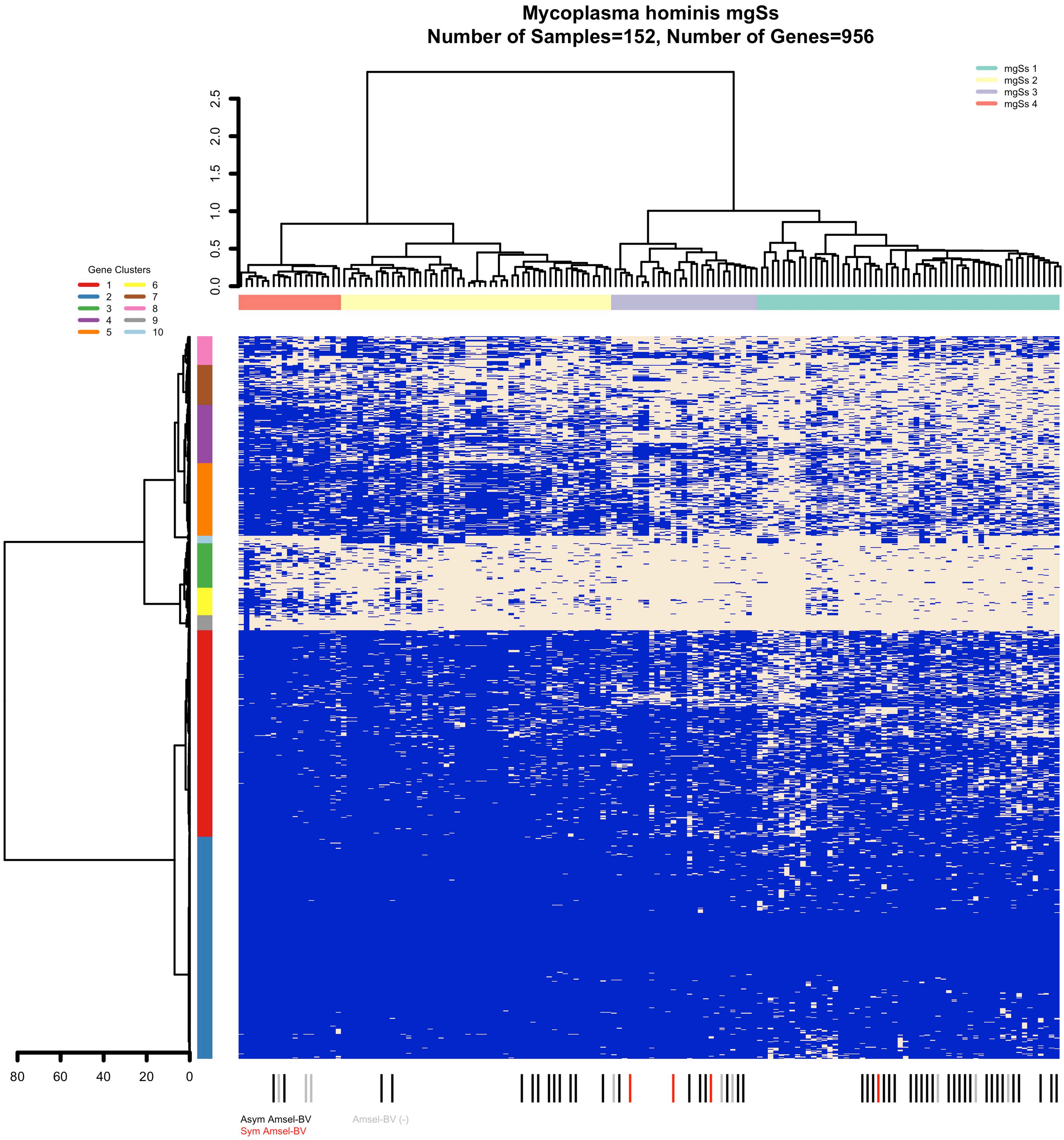
Mycoplasma hominis mgSs
Number of Samples=152, Number of Genes=956
mgSs 1
mgSs 2
mgSs 3
mgSs 4
Gene Clusters
1
2
3
4
5
6
7
8
9
10
0.0
0.5
1.0
1.5
2.0
2.5
80
60
40
20
0
Asym Amsel-BV
Sym Amsel-BV
Amsel-BV (-)

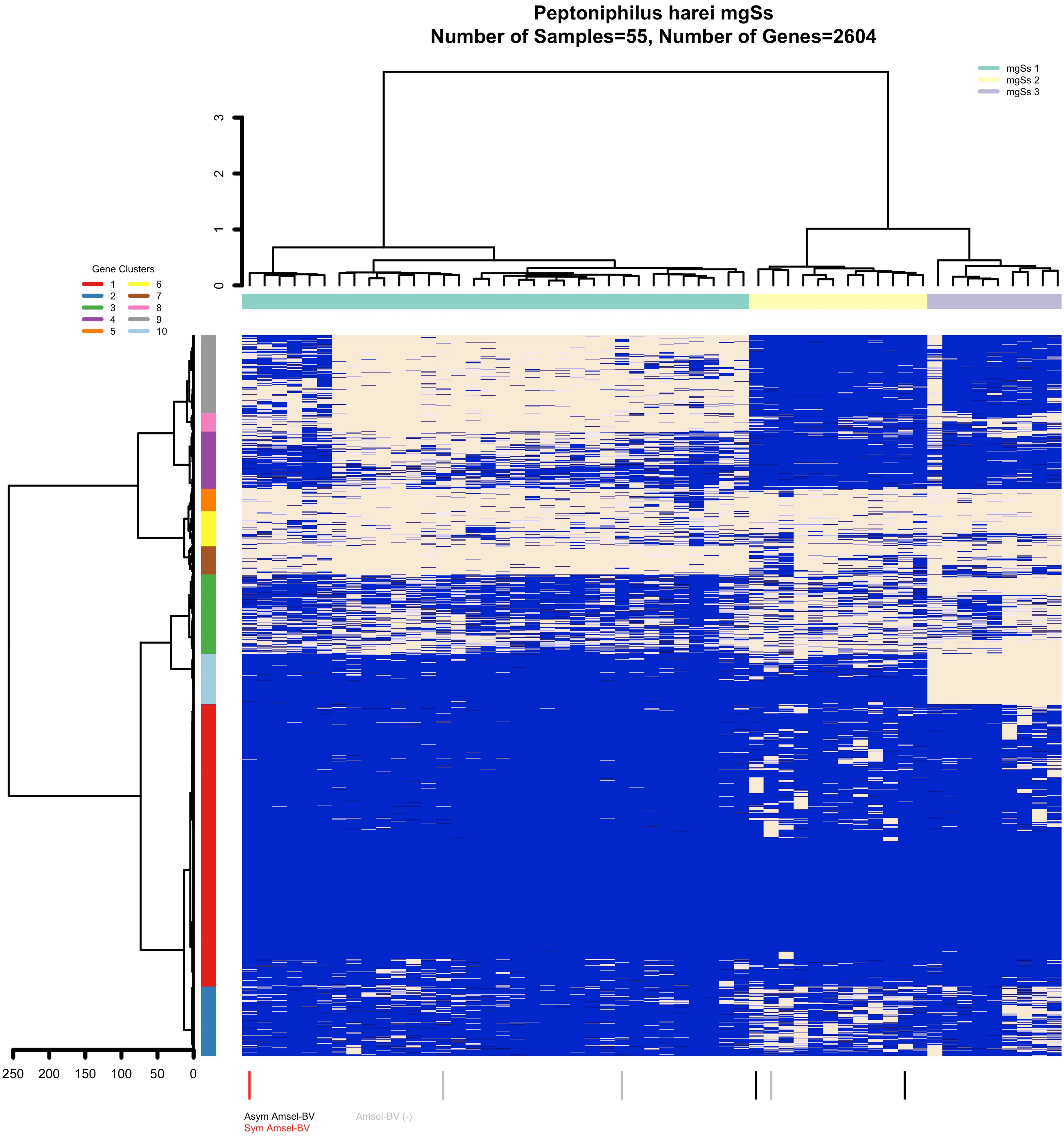
Peptoniphilus harei mgSs
Number of Samples=55, Number of Genes=2604
mgSs 1
mgSs 2
mgSs 3
Gene Clusters
1
2
3
4
5
6
7
8
9
10
0
1
2
3
250
200
150
100
50
0
Asym Amsel-BV
Sym Amsel-BV
Amsel-BV (-)

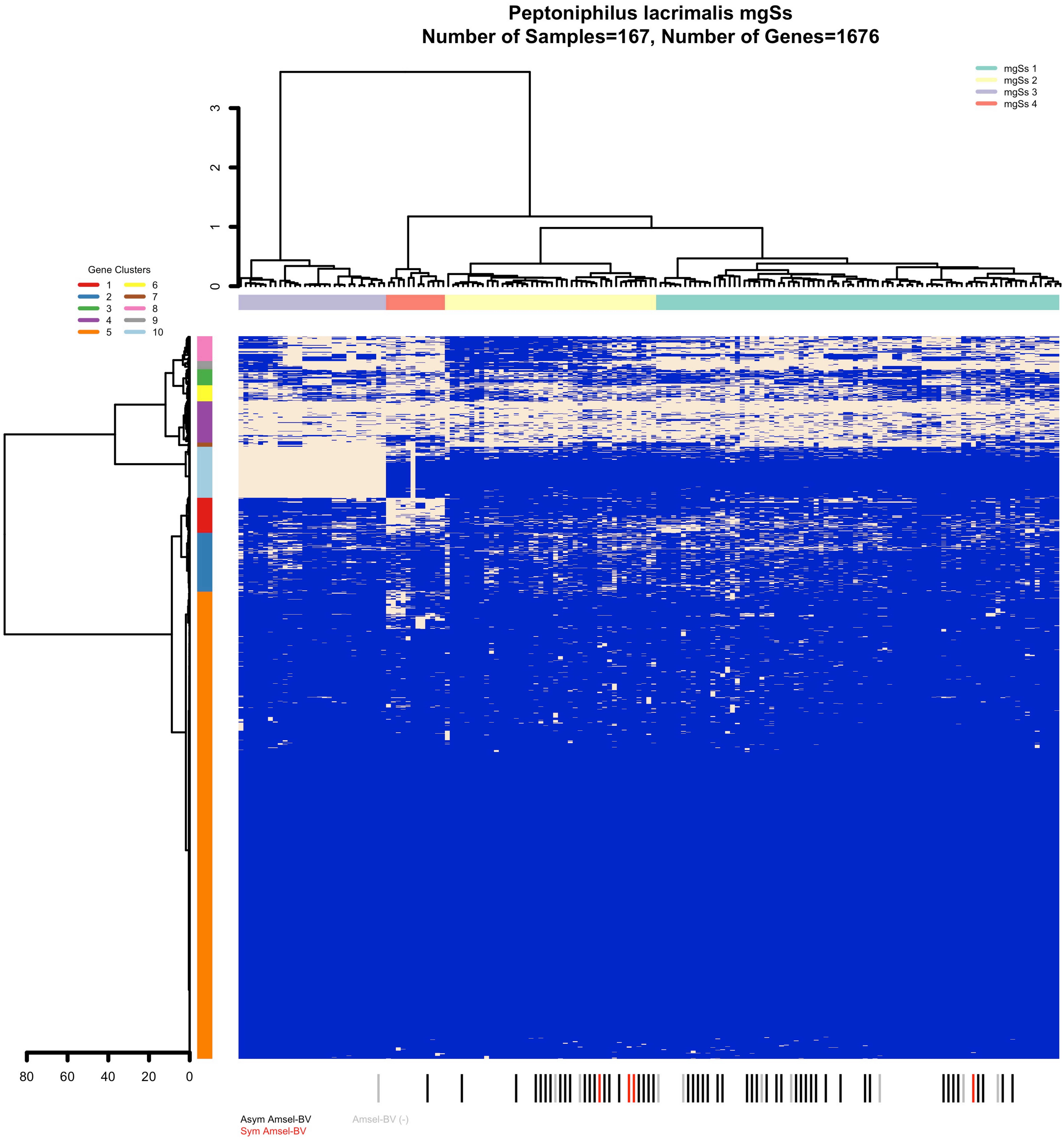

Peptoniphilus lacrimalis mgSs
Number of Samples=167, Number of Genes=1676
mgSs 1
mgSs 2
mgSs 3
mgSs 4
Gene Clusters
1
2
3
4
5
6
7
8
9
10
0
1
2
3
80
60
40
20
0
Asym Amsel-BV
Sym Amsel-BV
Amsel-BV (-)

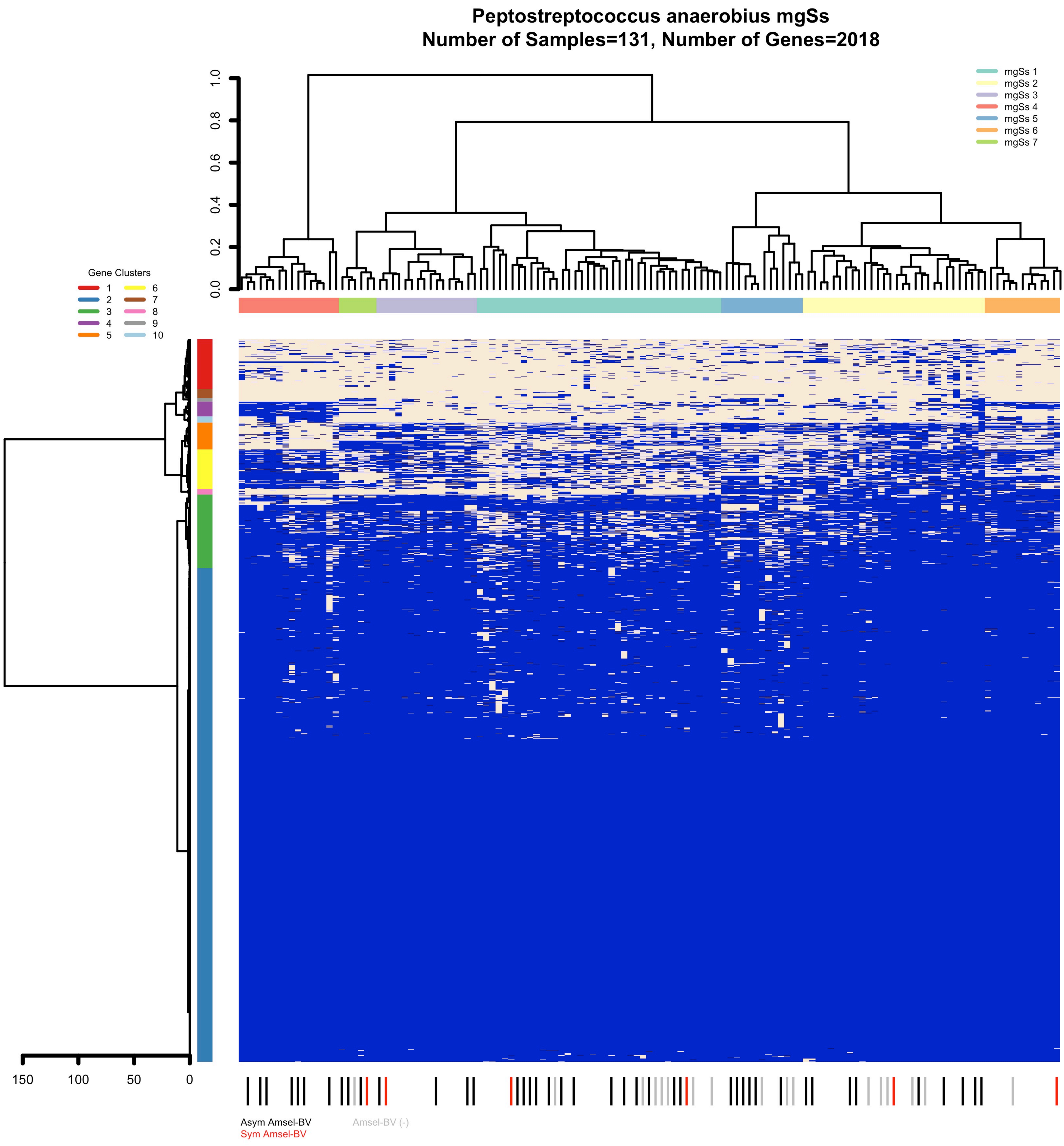
Peptostreptococcus anaerobius mgSs
Number of Samples=131, Number of Genes=2018
mgSs 1
mgSs 2
mgSs 3
mgSs 4
mgSs 5
mgSs 6
mgSs 7
Gene Clusters
1
2
3
4
5
6
7
8
9
10
0.0
0.2
0.4
0.6
0.8
1.0
150
100
50
0
Asym Amsel-BV
Sym Amsel-BV
Amsel-BV (-)

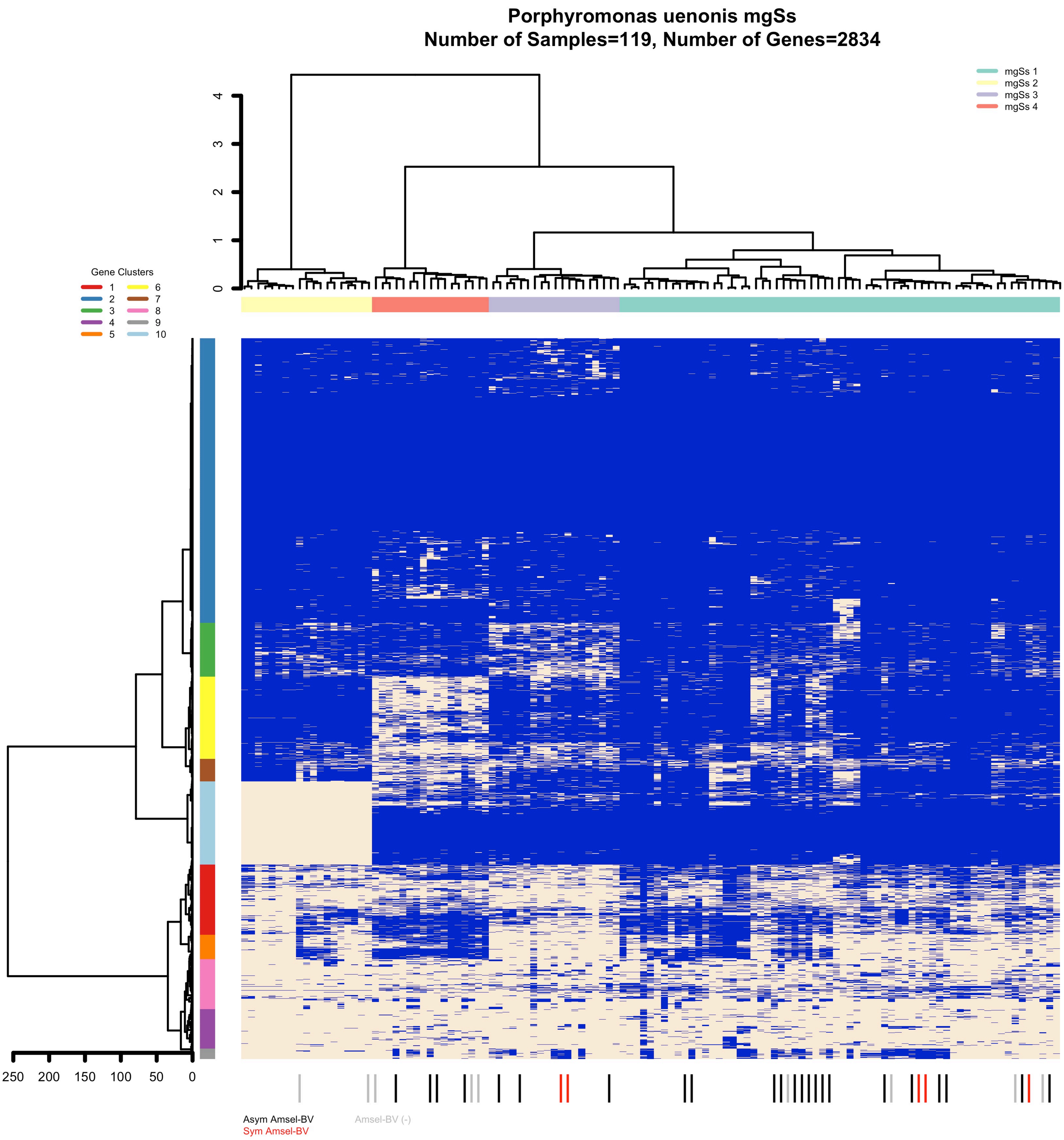
Porphyromonas uenonis mgSs
Number of Samples=119, Number of Genes=2834
mgSs 1
mgSs 2
mgSs 3
mgSs 4
Gene Clusters
1
2
3
4
5
6
7
8
9
10
0
1
2
3
4
250
200
150
100
50
0
Asym Amsel-BV
Sym Amsel-BV
Amsel-BV (-)

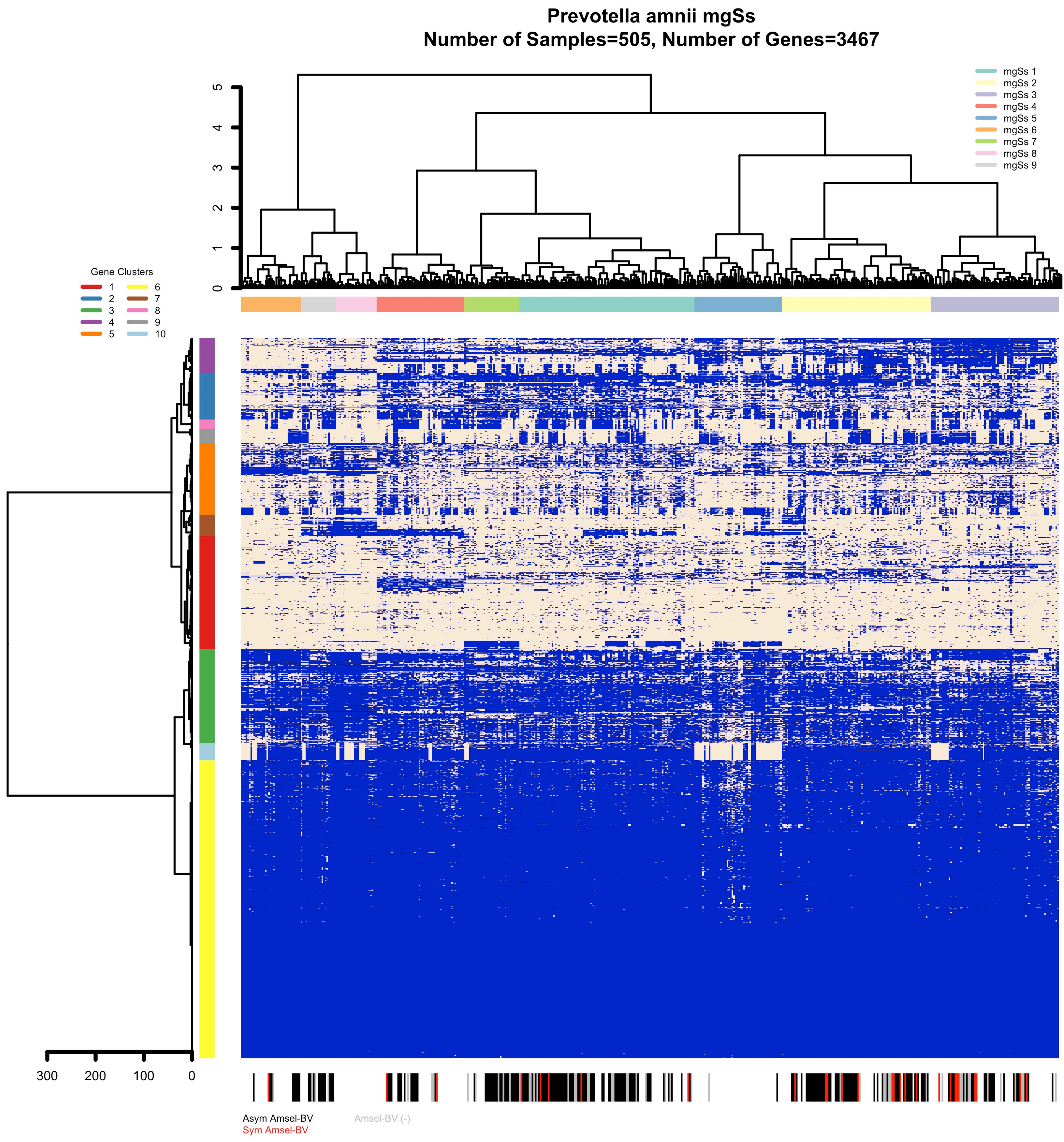

Prevotella amnii mgSs
Number of Samples=505, Number of Genes=3467
mgSs 1
mgSs 2
mgSs 3
mgSs 4
mgSs 5
mgSs 6
mgSs 7
mgSs 8
mgSs 9
Gene Clusters
1
2
3
4
5
6
7
8
9
10
0
1
2
3
4
5
300
200
100
0
Asym Amsel-BV
Sym Amsel-BV
Amsel-BV (-)

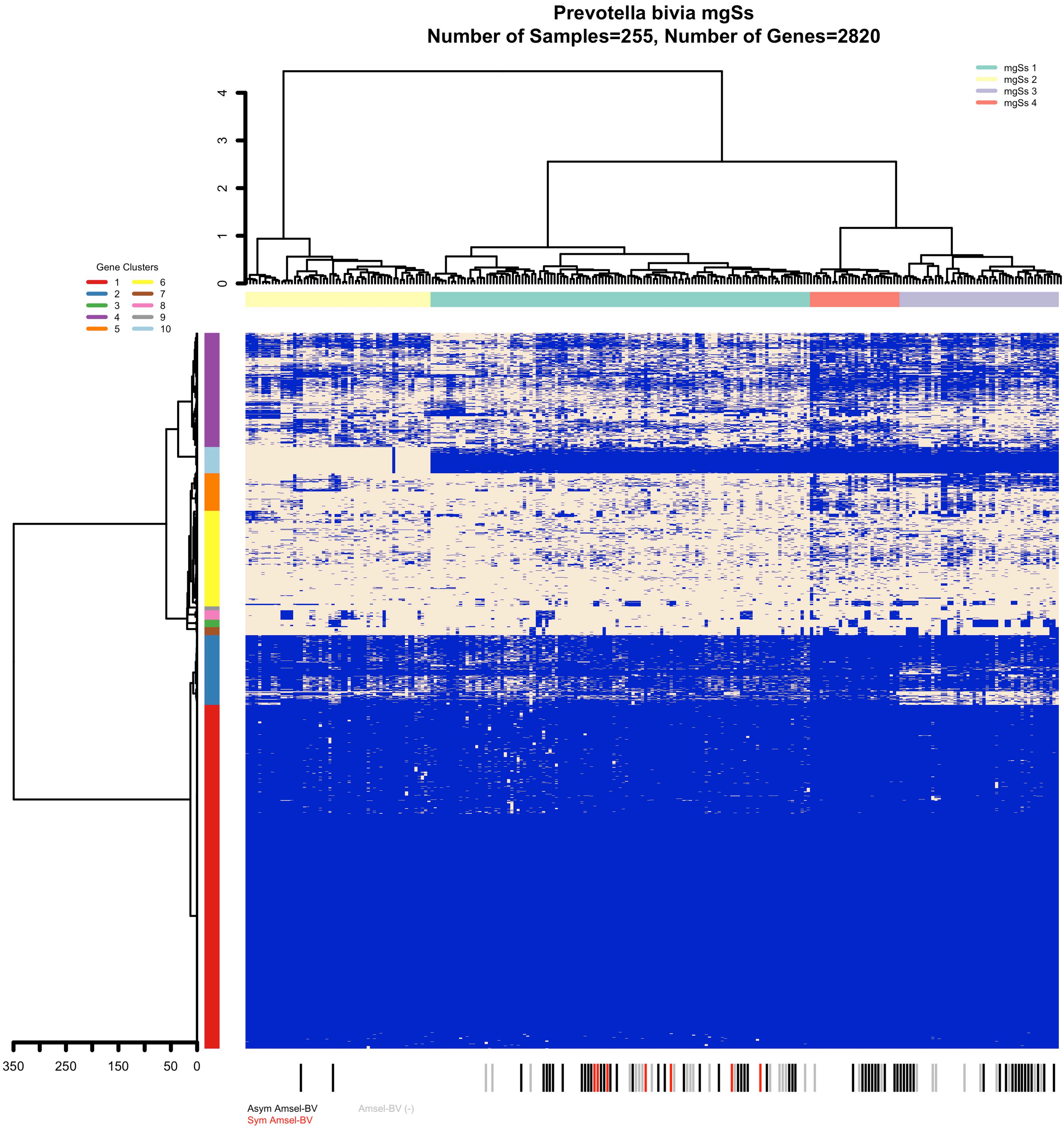
Prevotella bivia mgSs
Number of Samples=255, Number of Genes=2820
mgSs 1
mgSs 2
mgSs 3
mgSs 4
Gene Clusters
1
2
3
4
5
6
7
8
9
10
4
3
2
1
0
350
250
150
50
0
Asym Amsel-BV
Sym Amsel-BV
Amsel-BV (-)

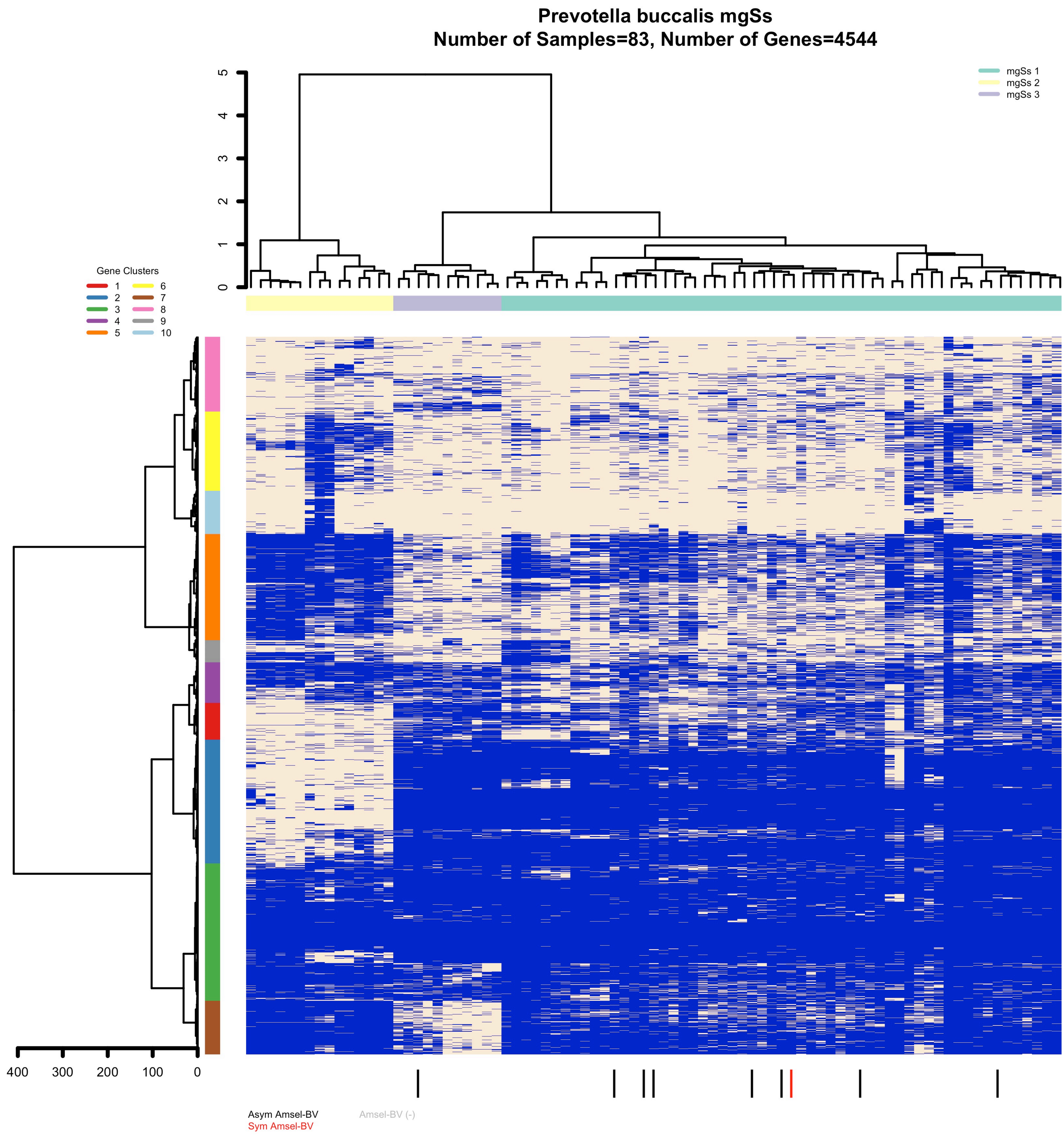

Prevotella buccalis mgSs
Number of Samples=83, Number of Genes=4544
mgSs 1
mgSs 2
mgSs 3
Gene Clusters
1
2
3
4
5
6
7
8
9
10
0
1
2
3
4
5
400
300
200
100
0
Asym Amsel-BV
Sym Amsel-BV
Amsel-BV (-)

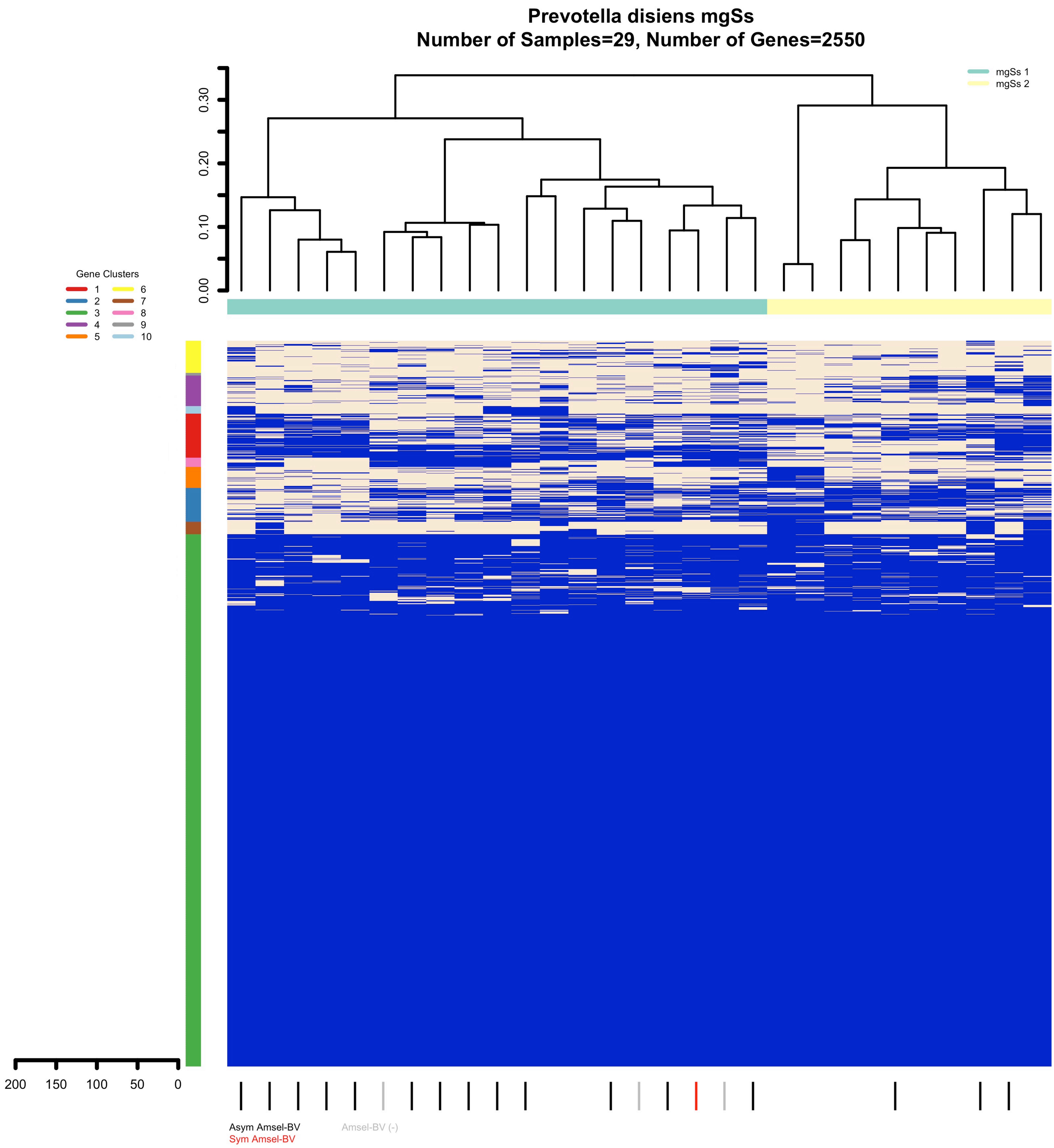
Prevotella disiens mgSs
Number of Samples=29, Number of Genes=2550
0.00
0.10
0.20
0.30
mgSs 1
mgSs 2
Gene Clusters
1
2
3
4
5
6
7
8
9
10
200
150
100
50
0
Asym Amsel-BV
Sym Amsel-BV
Amsel-BV (-)

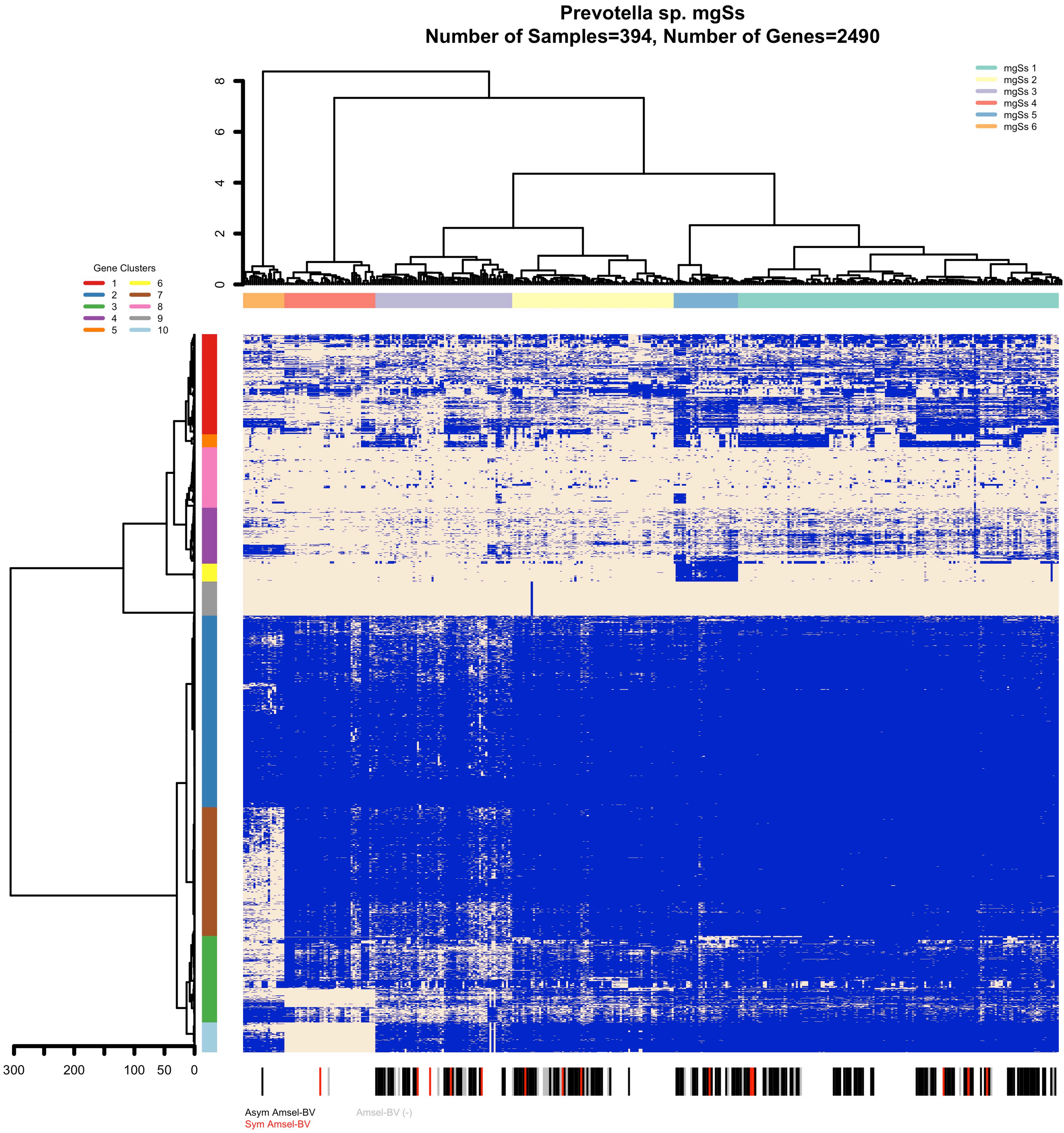

Prevotella sp. mgSs
Number of Samples=394, Number of Genes=2490
mgSs 1
mgSs 2
mgSs 3
mgSs 4
mgSs 5
mgSs 6
Gene Clusters
1
2
3
4
5
6
7
8
9
10
0
2
4
6
8
300
200
100
50
0
Asym Amsel-BV
Sym Amsel-BV
Amsel-BV (-)

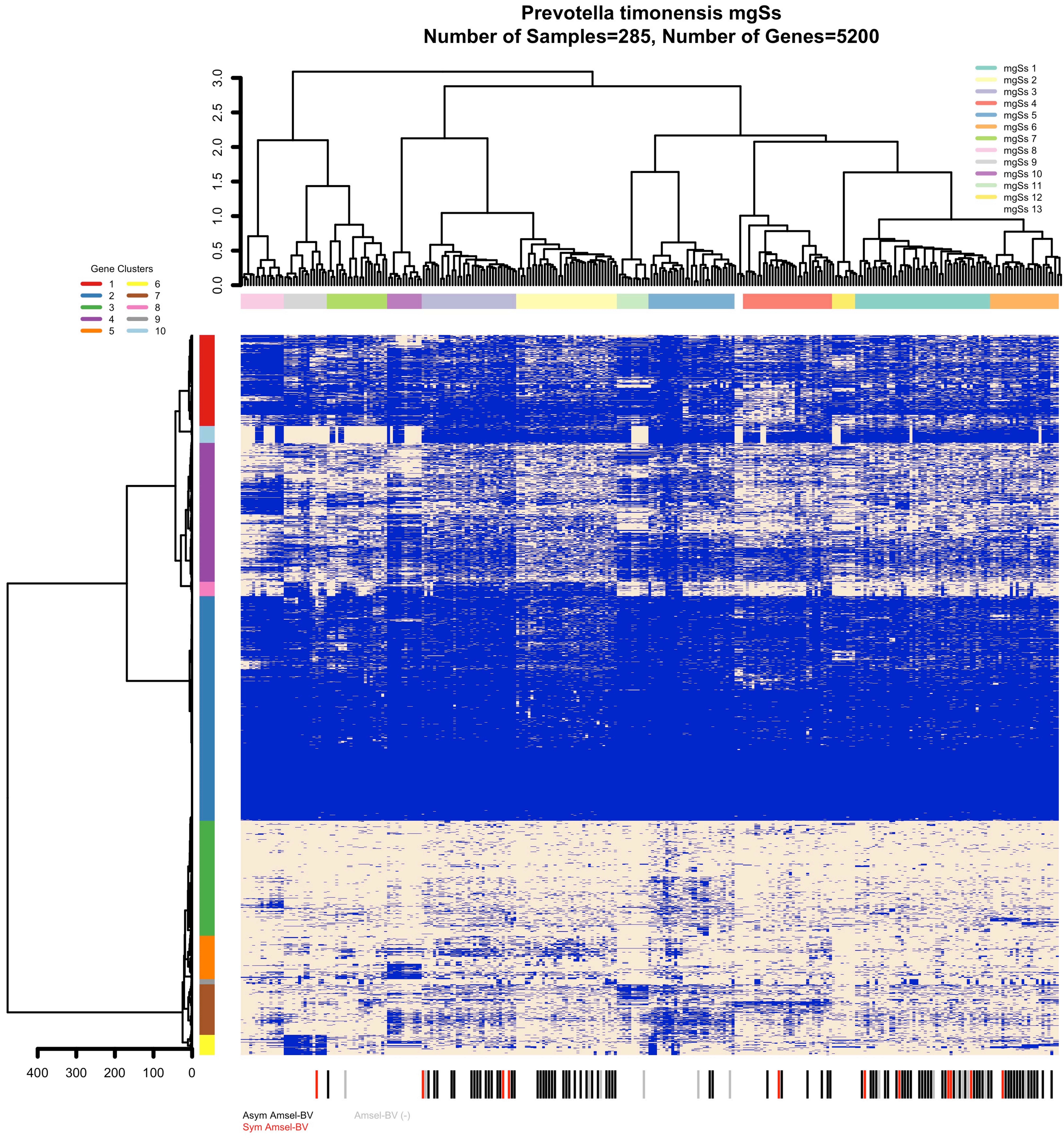
Prevotella timonensis mgSs
Number of Samples=285, Number of Genes=5200
0.0
0.5
1.0
1.5
2.0
2.5
3.0
mgSs 1
mgSs 2
mgSs 3
mgSs 4
mgSs 5
mgSs 6
mgSs 7
mgSs 8
mgSs 9
mgSs 10
mgSs 11
mgSs 12
mgSs 13
Gene Clusters
1
2
3
4
5
6
7
8
9
10
400
300
200
100
0
Asym Amsel-BV
Sym Amsel-BV
Amsel-BV (-)

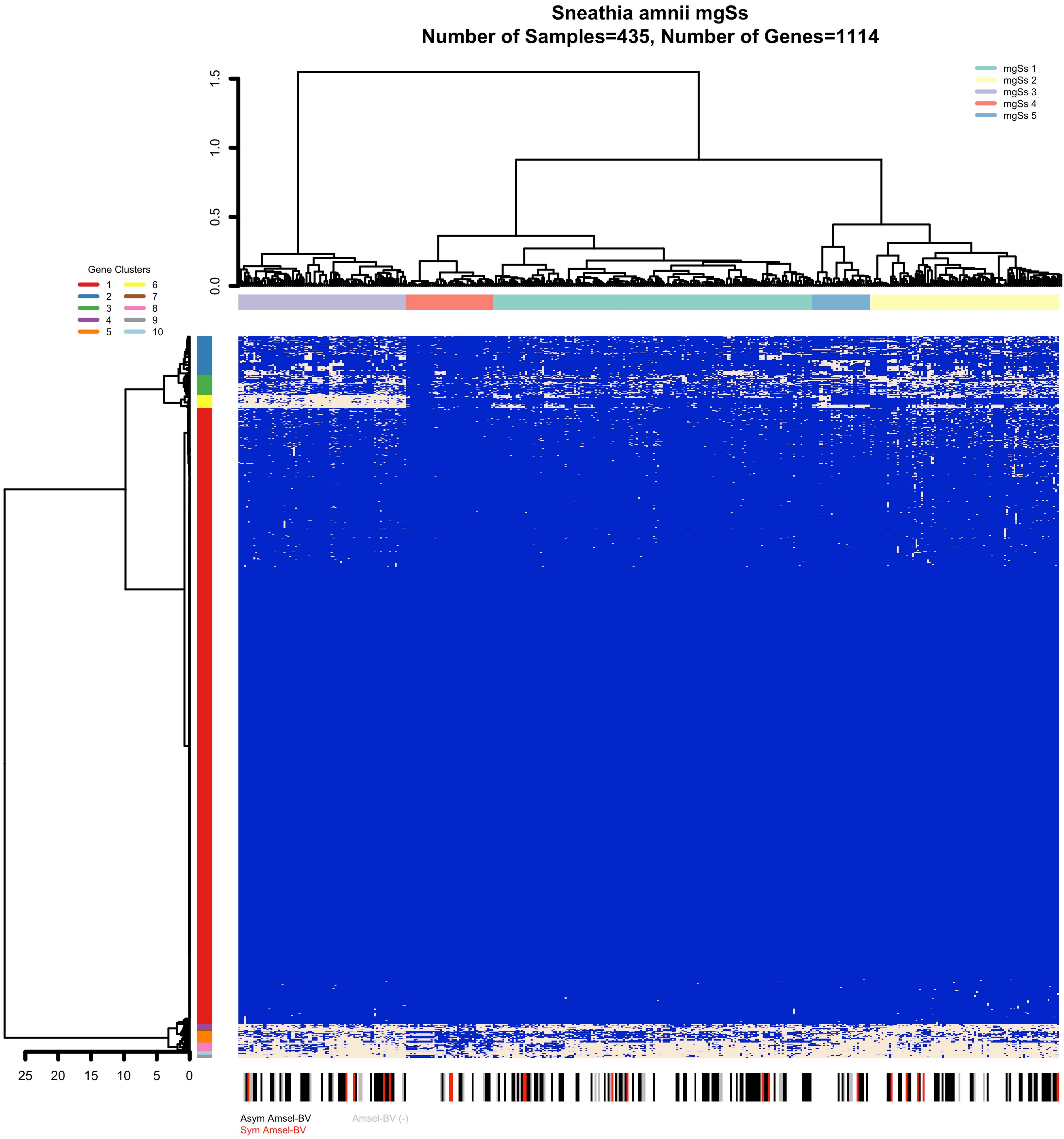

Sneathia amnii mgSs
Number of Samples=435, Number of Genes=1114
1.5
1.0
0.5
0.0
mgSs 1
mgSs 2
mgSs 3
mgSs 4
mgSs 5
Gene Clusters
1
2
3
4
5
6
7
8
9
10
25
20
15
10
5
0
Asym Amsel-BV
Sym Amsel-BV
Amsel-BV (-)

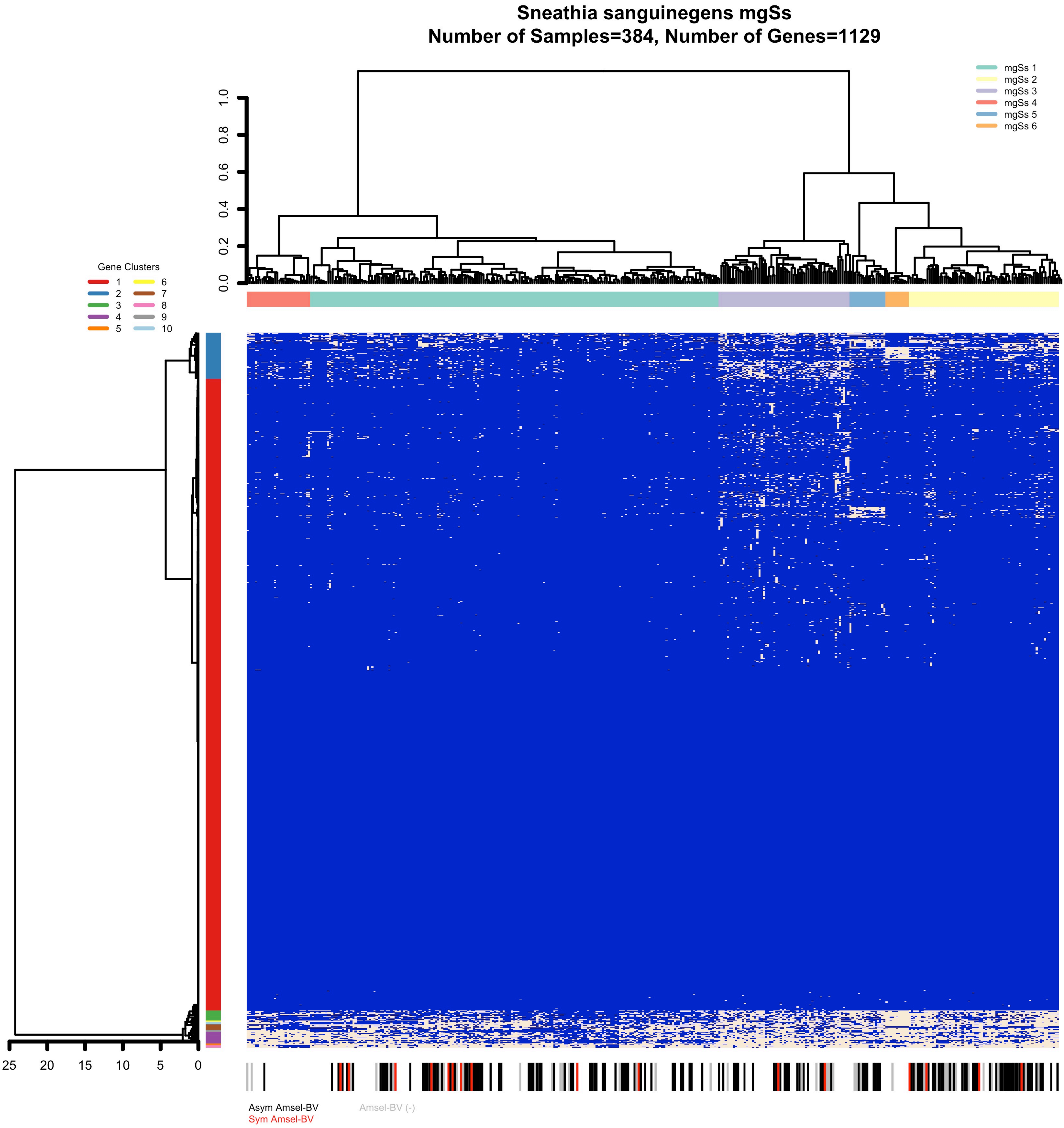
Sneathia sanguinegens mgSs
Number of Samples=384, Number of Genes=1129
mgSs 1
mgSs 2
mgSs 3
mgSs 4
mgSs 5
mgSs 6
Gene Clusters
1
2
3
4
5
6
7
8
9
10
1.0
0.8
0.6
0.4
0.2
0.0
25
20
15
10
5
0
Asym Amsel-BV
Sym Amsel-BV
Amsel-BV (-)

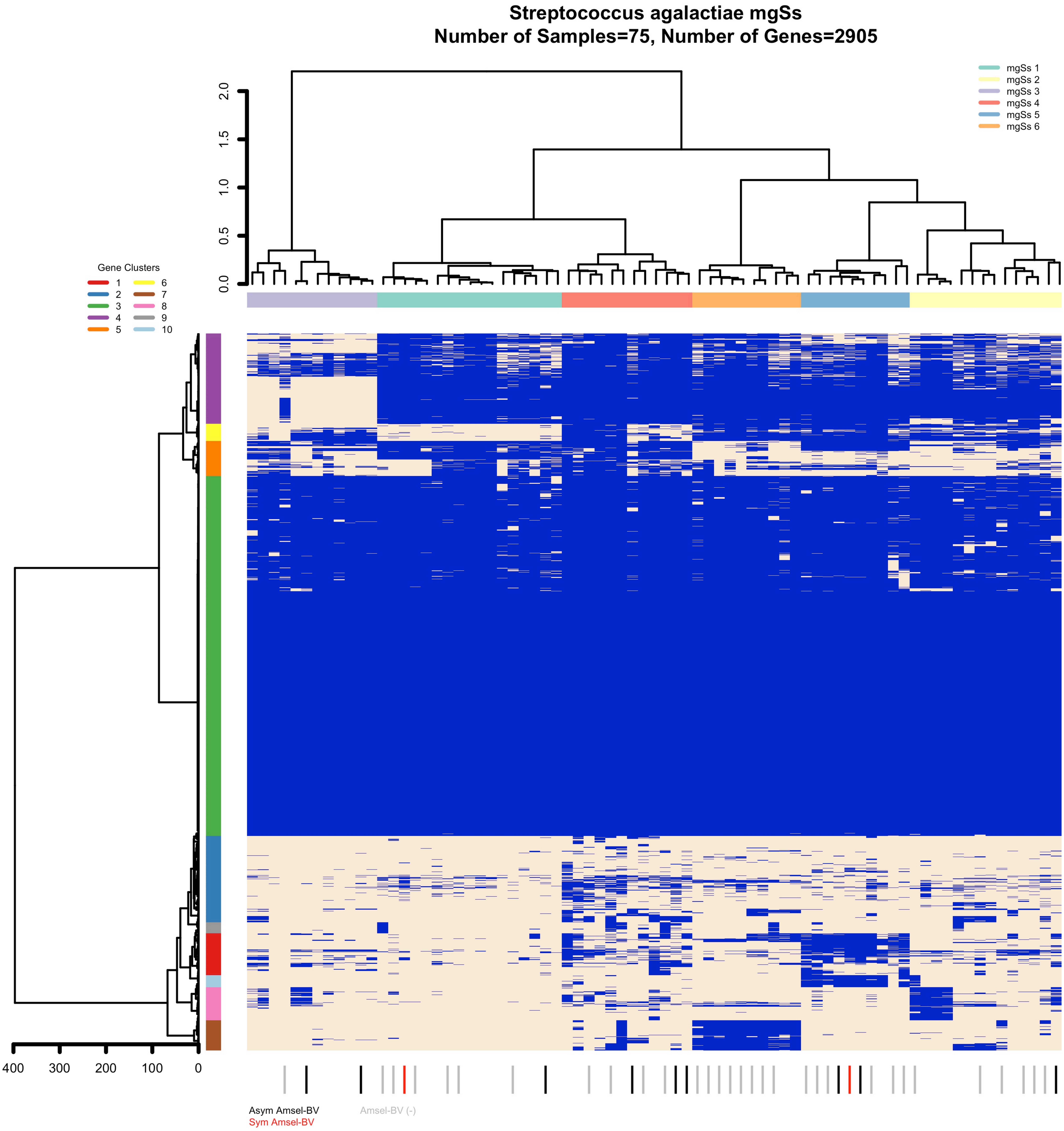
Streptococcus agalactiae mgSs
Number of Samples=75, Number of Genes=2905
mgSs 1
mgSs 2
mgSs 3
mgSs 4
mgSs 5
mgSs 6
Gene Clusters
1
2
3
4
5
6
7
8
9
10
0.0
0.5
1.0
1.5
2.0
400
300
200
100
0
Asym Amsel-BV
Amsel-BV (-)
Sym Amsel-BV

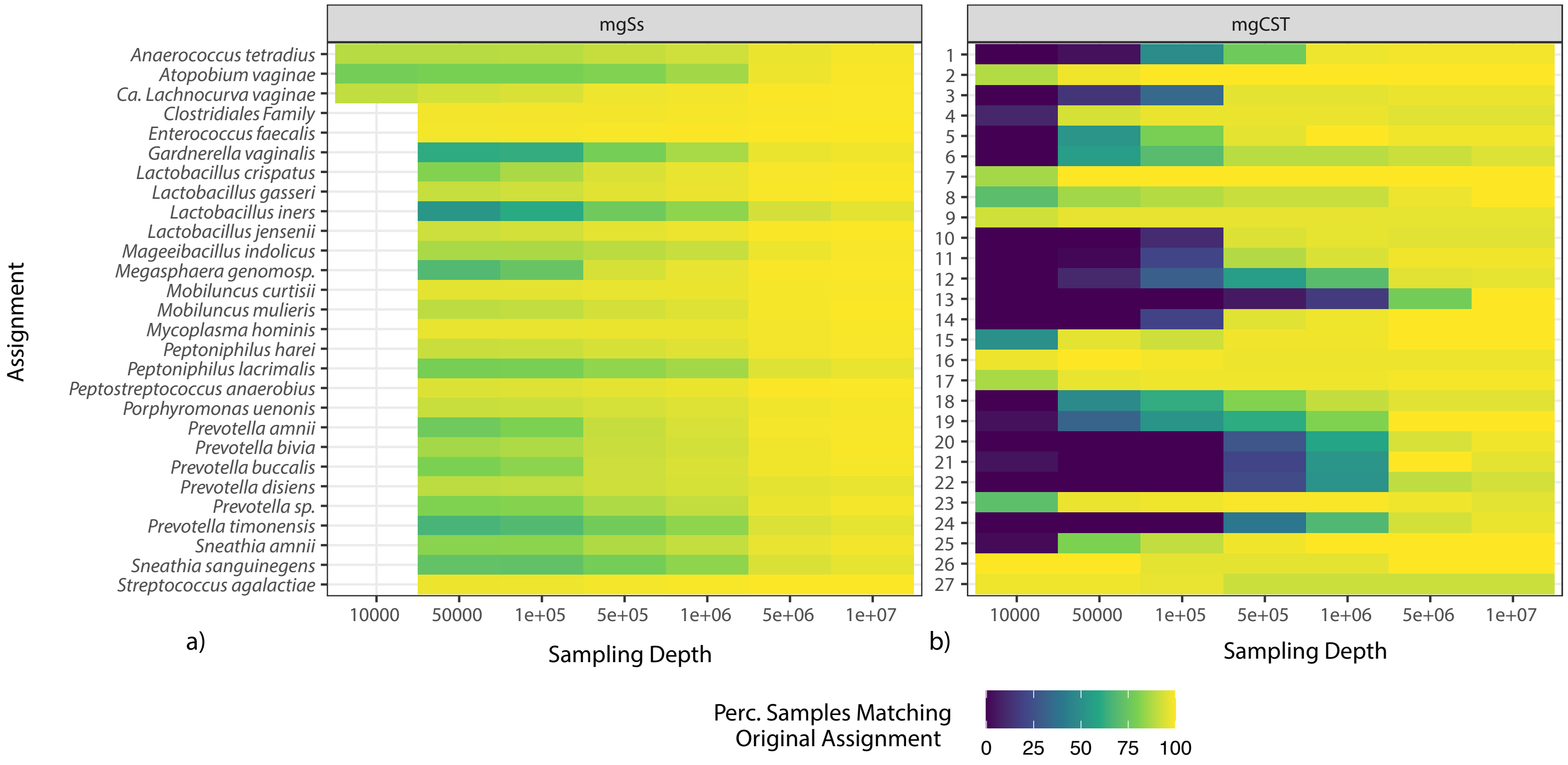

Fig. S10. Metagenomic subspecies (a, mgSs) and metagenomic community state types (b, mgCSTs) may be impacted with sampling (sequencing) depth. A minimum of 1 x 10^6 reads per sample is recommended for mgCST assignment.
